# Up-Regulation of Mitochondrial Activity and Acquirement of Brown Adipose Tissue-Like Property in the White Adipose Tissue of *Fsp27* Deficient Mice

**DOI:** 10.1371/journal.pone.0002890

**Published:** 2008-08-06

**Authors:** Shen Yon Toh, Jingyi Gong, Guoli Du, John Zhong Li, Shuqun Yang, Jing Ye, Huilan Yao, Yinxin Zhang, Bofu Xue, Qing Li, Hongyuan Yang, Zilong Wen, Peng Li

**Affiliations:** 1 Institute of Molecular and Cell Biology, Singapore, Singapore; 2 Department of Biological Sciences and Biotechnology, Protein Science Laboratory of Ministry of Education, Tsinghua University, Beijing, China; 3 Department of Biology, Hong Kong University of Science and Technology, Clear Water Bay, Hong Kong; 4 Department of Pathology, Fourth Military Medical University, Xi'an, China; 5 School of Biotechnology and Biomolecular Sciences, University of New South Wales, Sydney, Australia; 6 Department of Biochemistry, Hong Kong University of Science and Technology, Clear Water Bay, Hong Kong; Karolinska Institutet, Sweden

## Abstract

Fsp27, a member of the Cide family proteins, was shown to localize to lipid droplet and promote lipid storage in adipocytes. We aimed to understand the biological role of Fsp27 in regulating adipose tissue differentiation, insulin sensitivity and energy balance. *Fsp27* ^−/−^ mice and *Fsp27/lep* double deficient mice were generated and we examined the adiposity, whole body metabolism, BAT and WAT morphology, insulin sensitivity, mitochondrial activity, and gene expression changes in these mouse strains. Furthermore, we isolated mouse embryonic fibroblasts (MEFs) from wildtype and *Fsp27* ^−/−^ mice, followed by their differentiation into adipocytes *in vitro*. We found that Fsp27 is expressed in both brown adipose tissue (BAT) and white adipose tissue (WAT) and its levels were significantly elevated in the WAT and liver of *leptin*-deficient *ob/ob* mice. *Fsp27* ^−/−^ mice had increased energy expenditure, lower levels of plasma triglycerides and free fatty acids. Furthermore, *Fsp27* ^−/−^ *and Fsp27/lep* double-deficient mice are resistant to diet-induced obesity and display increased insulin sensitivity. Moreover, white adipocytes in *Fsp27* ^−/−^ mice have reduced triglycerides accumulation and smaller lipid droplets, while levels of mitochondrial proteins, mitochondrial size and activity are dramatically increased. We further demonstrated that BAT-specific genes and key metabolic controlling factors such as FoxC2, PPAR and PGC1α were all markedly upregulated. In contrast, factors inhibiting BAT differentiation such as Rb, p107 and RIP140 were down-regulated in the WAT of *Fsp27* ^−/−^ mice. Remarkably, *Fsp27* ^−/−^ MEFs differentiated *in vitro* show many brown adipocyte characteristics in the presence of the thyroid hormone triiodothyronine (T3). Our data thus suggest that Fsp27 acts as a novel regulator *in vivo* to control WAT identity, mitochondrial activity and insulin sensitivity.

## Introduction

Obesity, representing excess amount of body fat, develops as a result of a positive energy balance when energy intake exceeds that of metabolic expenditure. Adipose tissues play crucial roles in the development of obesity, with white adipose tissue (WAT) functioning as an energy storage organ,while brown adipose tissue (BAT) as an energy consumption organ [1]. Morphologically, white adipocytes are characterized by a large unilocular lipid droplet that occupies the majority of the cytoplasmic space, while brown adipocytes contain multiple and relatively smaller lipid droplets. BAT also contains large numbers of mitochondria packed with regularly arranged cristae, a characteristic of high mitochondrial activity. White adipocytes, on the other hand, have fewer mitochondria and their cristae are more compact. Although WAT and BAT both express a set of genes that regulates lipolysis, fatty acid metabolism, triacylglyceride (TAG) storage and insulin sensitivity [2], [3], BAT is known to be functionally more important as a thermogenic tissue [4]. It expresses a unique protein, uncoupling protein 1 (Ucp1), which functions to uncouple oxidative phosphorylation and converting this proton gradient energy into heat to maintain normal body temperature. Besides Ucp1, proteins such as type 2 iodothyronine deiondinase (Dio2) [5] and Cidea [6] have been shown to be highly enriched in BAT. PGC-1α [7], [8], a coactivator of multiple transcription factors such as nuclear respiratory factors (NRF-1/2) [9], [10] and peroxisome proliferator-activated receptor (PPAR) [11], is also highly expressed in BAT but low in WAT [12]. Furthermore, PGC-1α has been shown to coordinate multiple physiological cues for mitochondrial biogenesis and activity [13].

The exact origin of BAT and WAT and their developmental relationship is not clear. Both BAT and WAT are generally considered to be derived from a common preadipocyte pool [14]. Whereas recent report by Timmoms et al [15] suggest that brown and white preadipocytes may have different origins. BAT is apparently more closely related to muscle as brown preadipocytes appear to express myogenic specific proteins. Interestingly, differentiated WAT has been shown to acquire certain BAT-like properties and can be converted into an energy consumption organ under special conditions. It has been observed that cold-exposure or administration of β3-agonist could induce the emergence of multilocular brown adipocytes that contain high amount of mitochondria and expresses Ucp1 in WAT depots [16], [17]. Furthermore, ablation of β3-adrenoceptor diminishes the formation of BAT in white fat in cold exposed mice [18], suggesting the requirement of β-adrenoceptor signaling for the transdifferentiation of WAT to BAT. A similar increase in the occurrence of BAT in WAT areas was also observed in different strains of inbred mice [19]. Mice harboring increased BAT-like depots show a modest increase in β-adrenoreceptor signaling, which is sufficient to induce Ucp1 in these BAT-like depots. The requirement for PPARγ in acquiring BAT-like and energy consuming organ in WAT was also shown in PPARγ mutant (P465L) mice in which its WAT is unable to be converted to BAT in cold acclimatized mice [20]. On the other hand, attainment of fatty acid-oxidizing BAT-like cells in the WAT was also observed in several transgenic and knock-out mouse models. Transgenic knock-in mouse that replaces the C/EBPα gene with C/EBPβ gene (denoted as C/EBP β/β) was shown to lead to an increase in the expression of Gsα subunit, cAMP accumulation, enhanced mitochondrial biogenesis and Ucp1 expression in WAT [21]. WAT in mice with an overexpression of FoxC2 acquired certain BAT-like properties with increased expression levels of PGC1, Ucp1 and cAMP pathway proteins such as β3-adrenoceptor and the protein kinase A alpha regulatory subunit1 [22]. Similarly, in mice lacking negative regulators for BAT differentiation like *p107*, *Rb* and *RIP140*, there is a uniform replacement of WAT with BAT and elevated levels of PGC1α and Ucp1 [23], [24]. Although many factors and regulatory pathways have been shown to play important roles in maintaining WAT identity, upstream signals or factors that determine the fate of BAT vs WAT and those that initiate the conversion of WAT to BAT remain unclear.

Cide proteins including Cidea, Cideb and Fat specific protein 27 (Fsp27, also known as Cidec in human), share homology with the DNA fragmentation factor DFF40/45 at the N-terminal region [25]. Our previous work demonstrated that *Cidea* is expressed at high levels in BAT[6], whereas *Cideb* is expressed at high levels in liver [26]. Mice with deficiency in both *Cidea* and *Cideb* have higher energy expenditure, enhanced insulin sensitivity and are resistance to high-fat-diet (HFD)-induced obesity and diabetes [6], [26], suggesting a universal role of Cide proteins in the regulation of energy homeostasis. Fsp27 was originally identified in differentiated TA1 adipocytes and its expression is regulated by C/EBP (or C/EBP like) transcription factor [27]. Fsp27 mRNA is detected at high levels in WAT and moderate levels in BAT [6]. Lower levels of Fsp27 mRNA are present in skeletal muscle (SM) and lung [28]. In human, it is also detected in heart and many other tissues [29]. Furthermore, Fsp27 protein is detected in the lipid droplet enriched fraction [30] and over-expressed Fsp27 protein was shown to be targeted to lipid droplets and promotes TAG storage [31]. Recently, Fsp27 was also found to be a direct mediator of PPARγ-dependent hepatic steatosis [28]. However, its physiological role in regulating adipocyte function and the development of obesity is largely unknown. Here, we observed that Fsp27 protein was detected in both BAT and WAT, was dramatically up-regulated in the WAT and liver of *ob/ob* mice suggesting that Fsp27 expression is positively correlated with the development of obesity. *Fsp27* deficiency results in dramatically reduced WAT depot and the acquisition of a brown fat-like morphology in these WAT, typified by the appearance of smaller lipid droplets, increased mitochondrial size and their activity. Furthermore, both *Fsp27* deficient and *Fsp27/leptin* double deficient mice display improved insulin sensitivity and a lean phenotype. Genes such as PGC1α, Ucp1 and Dio2 that are normally expressed in BAT were expressed at high levels in the WAT of *Fsp27* **^−/−^** mice. Furthermore, regulators that maintain BAT identity like FoxC2 which was known to promote BAT differentiation was upregulated, whereas factors that inhibit BAT differentiation such as Rb, p107 and RIP140 were down-regulated in WAT of *Fsp27* **^−/−^** mice. Finally, differentiated mouse embryonic fibroblasts (MEFs) isolated from *Fsp27* **^−/−^** mice showed an increased propensity to acquire brown adipocyte characteristics, which is consistent with the *in vivo* observations in WAT of the mutant mice.

## Results

### Fsp27 is highly expressed in adipose tissue and up-regulated in *ob/ob* mice

**Figure 1:**
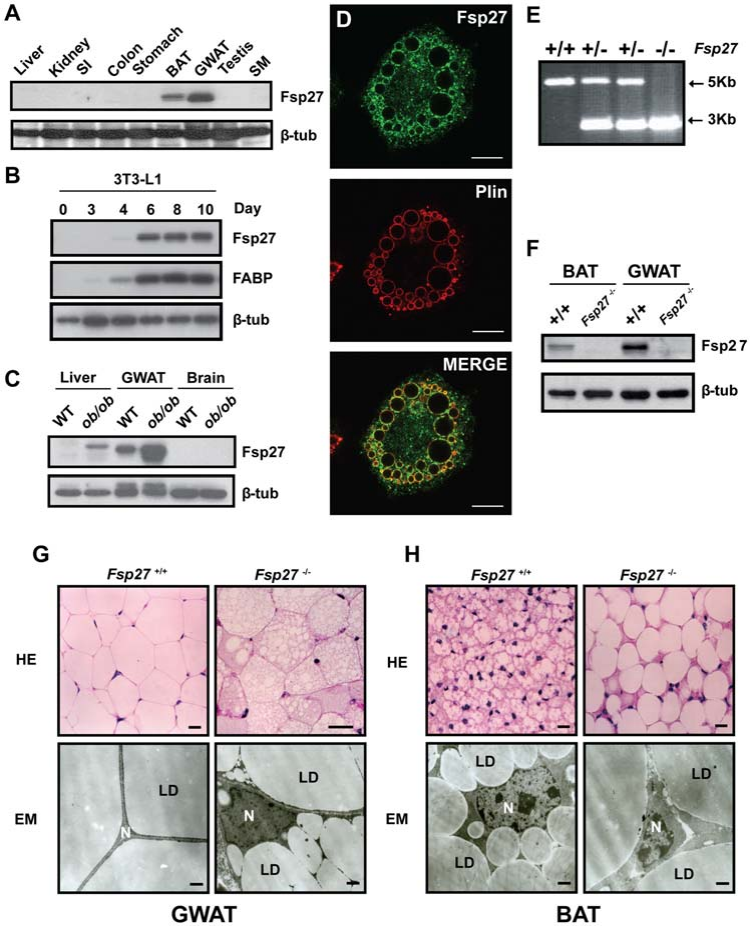
Fsp27 expression in mice tissue and the morphology of WAT and BAT in *Fsp27* ^−/−^ mice. A. Tissue distributions of Fsp27 protein by western blot analysis. SI, small intestine; BAT, brown adipose tissue; GWAT, gonadal white adipose tissue; SM, skeletal muscle. β-tubulin was used as a loading control. B. Fsp27 is expressed in differentiated 3T3-L1 adipocytes. FABP (fatty acid binding protein) is used as an adipocyte differentiation marker. Day 0 represents undifferentiated 3T3-L1 fibroblasts. . C. Elevated Fsp27 protein levels in the WAT and liver of leptin deficient (*ob/ob*) mice. WT, wild type. D. Indirect immunofluorescence showing co-localization of Fsp27 with perilipin (Plin) on lipid droplet surface in Day 8 post differentiated 3T3-L1 cells. E & F. Genotype analyses by PCR (E) and western blot (F) assays using tail genomic DNA and gonadal white adipose tissue (GWAT) lysate, respectively. +/+, +/−, −/− represent wild-type, *Fsp27* heterozygous and homozygous mice, respectively. G & H. Morphology of WAT and BAT of wild type and *Fsp27* ^−/−^ mice. HE, hemotoxylin and eosin staining. EM, electron microscope image. 3 months old mice were used for the experiments. LD, lipid droplets; N, nuclei. Scale bar = 25 µm and 1 µm for HE and EM, respectively. .

To determine the precise tissue distribution of Fsp27, we generated polyclonal antibody against Fsp27 and evaluated its expression profile by western blot analysis. High levels of Fsp27 protein were detected in WAT and moderate levels in BAT. No Fsp27 protein was detected in other tissues such as liver, kidney, colon and skeletal muscle (SM) **(** **Fig. 1A** **)**. Fsp27 was also detected in NIH3T3 L1 adipocytes after 4 days differentiation and its levels remain high during the course of differentiation **(** **Fig. 1B** **)**. These data suggest that Fsp27 is rather exclusively expressed at high levels in adipose tissue including BAT and WAT. The specific expression of Fsp27 in adipose tissue prompted us to check whether levels of Fsp27 protein were correlated with the development of obesity by examining the levels of Fsp27 in the WAT of *leptin*-deficient *ob/ob* mice. As shown in **Fig. 1C**, levels of Fsp27 in WAT were dramatically elevated in *ob/ob* mice compared with that of wild type mice. Furthermore, higher levels of Fsp27 were detected in the liver of *ob/ob* mice that contains large amount of lipid droplets. Using Fsp27 antibody, we checked the sub-cellular localization of Fsp27 in differentiated 3T3-L1 cells and observed that a fraction of Fsp27 was co-localized with lipid droplet specific marker *Perilipin* (**Fig. 1D**), consistent with previous observation that overexpression of GFP-Fsp27 fusion protein is targeted to lipid droplet [31], [32]. These data suggest that Fsp27 may positively correlate with the development of obesity and the accumulation of lipid in WAT and hepatocytes.

To elucidate the *in vivo* function of Fsp27 in adipocyte differentiation and the development of obesity, we isolated *Fsp27* genomic DNA and generated *Fsp27*-null mice by homologous recombination. The translation initiation site ATG in exon 2 of the *Fsp27* gene was replaced with a *Neo* gene cassette that is transcribed by a PGK promoter from the opposite direction **(Fig. S1A)**. This strategy effectively deleted the first 31 amino acids of *Fsp27*. We confirmed the gene disruption by PCR and western blot analyses. Genetic deletion generates a PCR product of a 3 Kb instead of a 5 Kb wild-type band (**Fig. 1E**). Western blot analysis also showed that no Fsp27 protein was detected in either WAT or BAT of *Fsp27* ^−/−^mice, suggesting that *Fsp27* gene was successfully disrupted (**Fig. 1F**).

To investigate the role of Fsp27 in adipocyte differentiation, we first examined the morphology of fat pads. White fat pads from various locations such as gonadal fat and subcutaneous fat from *Fsp27* ^−/−^ mice are all significantly reduced in sizes compared with those from wild type mice. Furthermore, WAT of *Fsp27* ^−/−^ mice isolated from the gonadal region had a dark-red color and abundant blood vessel circulation (data not shown). Staining by hemotoxylin and eosin (HE) revealed that white adipocytes from gonadal region of *Fsp27* ^−/−^ mice had small and multiple lipid droplets, whereas white adipocytes of wild type mice had large and unilocular lipid droplet (**Fig. 1G**). Electron microscope (EM) analysis using semi-thin sections of the WAT showed that white adipocytes of *Fsp27* ^−/−^ mice had defined minuscule lipid droplets distinguishable from each other, and occupied the cytosol like in brown adipocytes (**Fig. 1G**). We also noted that the morphological features were homogeneous throughout the entire WAT. As Fsp27 is also expressed in BAT, we next compared the morphology of BAT of wild type and *Fsp27* ^−/−^ mice. The BAT dissected from *Fsp27* ^−/−^ mice appear to be pale and slightly enlarged compared with the wild type BAT. Histological examination of BAT from wild type and *Fsp27* **^−/−^** mice indicates that both contain multilocular lipid droplets. However, the sizes of lipid droplet in BAT of *Fsp27* ^−/−^ mice were significantly larger than that of wildtype mice. EM analysis confirmed such an observation (**Fig. 1H**). No gross morphological difference for SM, heart or liver was observed between wild type and *Fsp27 m*utant mice (data not shown). These data suggest that WAT of *Fsp27* **^−/−^** mice acquires a morphology similar to that of BAT with smaller and multiple lipid droplets and abundant blood vessel circulation.

### Lean phenotype in *Fsp27* ^−/−^ and *leptin/Fsp27* double deficient mice

**Figure 2:**
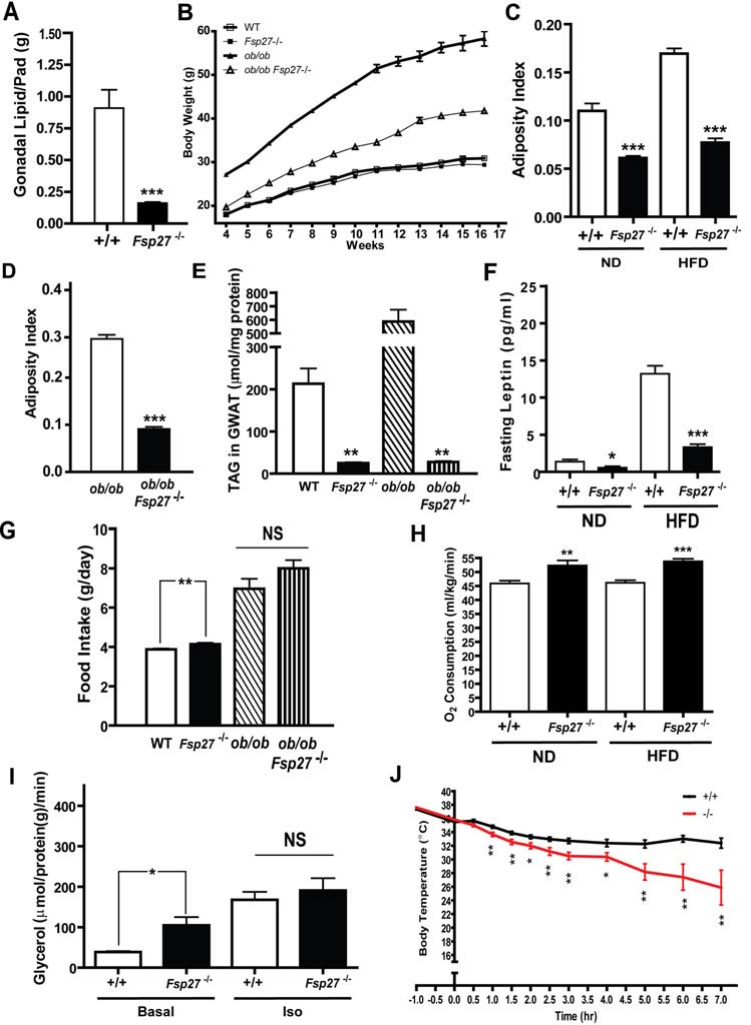
Lean phenotype of *Fsp27* deficient and *leptin*/*Fsp27* double deficient mice. A. Total lipid content of gonadal white fat pad from 3-month old wild-type (+/+) and *Fsp27* mutant (−/−) mice, showing a decrease in lipid content in the WAT of *Fsp27* ^−/−^ mice. 5 pairs of mice were used for analysis. ***p<0.001. B. Body weight of wild type (WT), *Fsp27* deficient (*Fsp27* ^−/−^), *leptin* deficient (*ob/ob)* and *leptin*/*Fsp27* double deficient (*ob/ob/Fsp27* ^−/−^ *)* mice. (wildtype: n = 12; *Fsp* ^−/−^: n = 12, *ob/ob*, n = 10; *ob/ob/ Fsp27^−/−^*, n = 11, P<0.001)C. Adiposity index of 10 months old wild-type (+/+) and *Fsp27* mutant mice (−/−) fed with a normal diet (ND) or high fat diet (HFD). Fsp27 mutant mice have significantly less adipose tissue. (ND: +/+ n = 13, −/− n = 14 , HFD: +/+ n = 12, −/− n = 15) (***p<0.001). D. Adiposity index of 3 months old *ob/ob* and *ob/ob*/*Fsp27^−/−^* mice. *ob/ob*: n = 8, *ob/ob/ Fsp27^−/−^*: n = 8) (***p<0.001). E. TAG content in gonadal white fat (GWAT) of wild type (WT), *Fsp27* mutant (*Fsp27* ^−/−^), *ob/ob,* and *ob/ob/ Fsp27^−/−^* mice fed with normal diet. 3 mice were used for each genotype. **p<0.01. F. Fasting serum leptin level in mice under normal diet (ND) or high-fat diet (HFD) feeding conditions. ND: +/+ n = 11, −/− n = 8; HFD: +/+ n = 14, −/− n = 11. *p<0.05, ***p<0.001. G. Increased food intake in *Fsp27* mutant and *ob/ob/Fsp27* ^−/−^ mice. Wild type: n = 15, *Fsp27* ^−/−:^ n = 17; *ob/ob*: n = 6, *ob/ob/Fsp27* ^−/−^: n = 6. **p<0.01. H. Whole body O_2_ consumption rate of wild-type or *Fsp27* mutant mice fed with a ND or HFD. ND: +/+ n = 14, −/− n = 13, HFD: +/+ n = 16, −/− n = 18. **p<0.01, ***p<0.001. I. Lipolysis rate in GWAT of 3 months old wild type (+/+) and Fsp27 mutant (−/−) mice. Basal refers to no isoproterenol (Iso,1 µM) treatment.. N = 4 for each genotype. *p<0.05. J. Core Body temperature of wild-type (+/+) or Fsp27 mutant (−/−) mice after animals were exposed to cold ( 4°C). −1 refers to core body temperature 1 hr before transfer of mice to 4°C. Fsp27 mutant mice had lower core body temperature when exposed to cold. (+/+ n = 25, −/− n = 20, *p<0.05, **p<0.01, ***p<0.001).

The drastic reduction of lipid droplet size in WAT prompted us to investigate if the atrophy of WAT was due to its loss of lipid content by measuring the total amount of lipid in gonadal WAT. We found that total lipid in gonadal white fat (GWAT) pad was reduced by almost 6 fold (from 0.8995±0.1436 g in wild type to 0.1512±0.0079 g in *Fsp27* ^−/−^ mice, P<0.001, **Fig. 2A** **)**, which strongly suggests that the reduction in fat pad size observed in WAT of *Fsp27* **^−/−^** mice is a result of decreased lipid content in the adipocytes. The total amount of protein and DNA content within this fat pad was similar between wild-type and *Fsp27* null mice (data not shown). We then investigated the potential role of *Fsp27* in regulating overall body weight and adiposity. Under normal diet (ND) conditions, the body weight of wild type and *Fsp27* ^−/−^ mice were similar (**Fig. 2B**), while the average body weight of *ob/ob* mice was dramatically higher than that of wild type or *Fsp27* ^−/−^ mice. However, the body weight of *leptin/Fsp27* double deficient (*ob/ob/Fsp27* ^−/−^ *)* mice was significantly lower than that of *ob/ob* mice (**Fig. 2B, P** **<0.001**), suggesting that Fsp27-deficiency could counteract with weight gain in *ob/ob* mice. When fed with a ND, the adiposity index of *Fsp27* ^−/−^ mice (0.0611±0.0021) was approximately 45% lower than that of wild type mice (0.1101±0.0008, P<0.0001). We also observed no difference in overall size with a slightly increased weight of some individual organs such as liver and heart in *Fsp27* ^−/−^ mice compared to control animals (**data not shown**). When mice were fed with a high fat diet (HFD), there was a 54% reduction in adiposity index in *Fsp27* null mice (0.0772±0.0042) compared to that of wild type mice (0.1695±0.0052, P<0.0001, **Fig. 2C**). While the adiposity index of *ob/ob* mice is approximately 30%, the adiposity index for *ob/ob/Fsp27^−/−^* mice (10%) was markedly lower (P<0.001), representing a 70% reduction in total body fat (**Fig. 2D**). Consistent with the decreased adiposity index, the weight of fat pads from different anatomical locations in *Fsp27^−/−^* as well as WAT and BAT in *ob/ob/Fsp27^−/−^* mice was significantly reduced (**Table 1** **&** **2** **)**. Levels of TAG in the WAT of *Fsp27* ^−/−^ mice (25.3±2.6 µmol/mg protein) were much lower than that of wild type mice (213.2±62.5 µmol/mg protein), representing a 90% reduction (P<0.01, **Fig. 2E**). Furthermore, TAG levels in the WAT of *ob/ob/Fsp27^−/−^* mice were much lower (27.7±3.9 vs 586.9±100.7 µmol/mg protein, P<0.01, **Fig. 2E**) compared with that of *ob/ob* mice. In addition to a reduced amount of TAG in WAT, the amount of TAG in the liver and skeletal muscle of *Fsp27* ^−/−^ mice was also significantly reduced (**Fig. S1B**). Therefore, the reduced TAG levels in WAT were not due to alternative lipid storage in other tissues such as liver and SM.

**Table 1:** Blood Chemistry and Fat Pad Weight of 10 months old wildtype (+/+) and *Fsp27^−/−^* (−/−) mice.

	Normal Diet					High Fat Diet				
	+/+		−/−			+/+		−/−		
	N	Mean±SEM	N	Mean±SEM	P	N	Mean±SEM	N	Mean±SEM	P
NEFA (mEq/l) (Fed)	8	1.014±0.243	8	0.635±0.150	***0.0021*** **	9	1.305±0.192	9	1.086±0.147	***0.0314*** *
NEFA (mEq/l) (Fast)	8	1.358±0.148	8	1.189±0.120	***0.0250*** *	9	1.561±0.277	9	1.120±0.283	***0.0012*** **
TG (mmol/l) (Fed)	8	0.560±0.162	8	0.463±0.056	0.0525	9	0.615±0.136	9	0.247±0.032	***<0.0001*** ***
TG (mmol/l) (Fast)	8	1.563±0.308	8	1.045±0.192	***<0.0001*** ***	9	2.335±0.277	9	1.314±0.254	***<0.0001*** ***
Ketone Bodies (mmol/L) (Fed)	14	0.070±0.025	12	0.035±0.016	***0.0003*** ***	8	0.036±0.01	14	0.066±0.026	***0.0010*** ***
Ketone Bodies (mmol/L) (Fast)	12	1.487±0.107	14	0.534±0.310	***<0.0001*** ***	16	0.730±0.44	18	0.515±0.295	0.1
Insulin (ng/ml) (Fed)	12	0.946±0.550	11	1.076±0.594	0.591	7	1.522±0.657	12	2.067±1.07	0.242
Insulin (ng/ml) (Fast)	#9	#0.357±#0.084	#7	#0.377±#0.166	0.7586	13	1.768±0.464	8	1.767±0.836	0.999
Leptin (pg/ml) (Fed)	12	2.720±1.732	12	1.419±1.023	***0.0350*** *	12	21.157±8.824	13	13.52±5.588	***0.0190*** *
Leptin (pg/ml) (Fast)	11	1.375±0.955	7	0.531±0.498	***0.0480*** *	14	13.198±4.091	11	3.329±1.328	***<0.0001*** ***
Glucose (mmol/l) (Fed)	16	11.70±2.635	17	11.753±4.479	0.9675	18	11.10±3.378	19	10.605±1.504	0.5651
Glucose (mmol/l) (Fast)	15	6.887±1.132	17	7.729±1.768	0.1244	18	10.717±1.827	19	11.968±1.995	0.0548
Inguinal Fat	15	0.023±0.009	14	0.013±0.002	***0.008*** **	13	0.039±0.011	15	0.017±0.004	***<0.0001*** ***
Gonadal Fat	15	0.024±0.006	14	0.004±0.001	***<0.0001*** ***	13	0.028±0.007	15	0.006±0.002	***<0.0001*** ***
Retroperitoneal Fat	15	0.014±0.004	14	0.008±0.002	***0.0001*** ***	13	0.023±0.01	15	0.017±0.007	0.07
Mesenteric Fat	15	0.014±0.003	14	0.009±0.002	***<0.0001*** ***	13	0.016±0.005	15	0.008±0.002	***<0.0001*** ***
Subcuteneous Fat	15	0.018±0.007	14	0.012±0.004	***0.009*** **	13	0.023±0.009	15	0.015±0.005	***0.012*** *

All values are means±s.e.m. Statistical analysis was done with two-tailed unpaired student t-test. N represents number of mice used.

# Data obtained from 3 months old mice.

* P<0.05,

** P<0.01,

*** P<0.001

**Table 2:** Blood Chemistry and Tissue Weight of *ob/ob* and *ob/ob/Fsp27* ^−/−^ mice

	*ob/ob*		*ob/ob*/*Fsp27* ^−/−^		
			
	N	Mean±SEM	N	Mean±SEM	P
NEFA (mEq/l) (Fed)	8	3.906±0.383	8	3.617±0.212	***0.2946***
NEFA (mEq/l) (Fast)	8	4.582±0.686	8	3.873±0.382	***0.01148*** *
TG (mg/l) (Fed)	8	0.3185±0.0142	8	0.3682±0.0216	***<0.0001*** ***
TG (mg/l) (Fast)	8	0.3584±0.0222	8	0.4256±0.0232	***0.0028*** **
Cholesterol (mmol/l) (Fed)	8	5.805±0.014	8	6.518±0.013	***0.016*** *
Cholesterol (mmol/l) (Fast)	8	5.517±0.020	8	6.147±0.007	**0.04748** *
Insulin (ng/ml) (Fed)	8	7.986±0.162	8	6.290±0.209	***<0.0001*** ***
Insulin (ng/ml) (Fast)	8	7.426±0.320	8	6.011±0.133	***<0.0001*** ***
Glucose (mmol/l) (Fed)	12	17.24 ± 1.129	12	16.76± 1.210	***0.3226***
Glucose (mmol/l) (Fast)	12	11.90 ± 1.369	12	11.37± 0.884	***0.2692***
Gonadal Fat Pad	10	0.061 ± 0.007	10	0.004 ± 0.002	***<0.0001*** ***
BAT	10	0.061 ± 0.007	10	0.004 ± 0.002	***<0.0001*** ***
Liver	10	0.102 ± 0.009	10	0.009 ± 0.002	***<0.0001*** ***

All values are means ± s.e.m. Statistical analysis was done with two-tailed unpaired student t-test. N represents number of mice used.

* P<0.05,

** P<0.01,

*** P<0.001

In accordance with the presence of lower amount of WAT, *Fsp27*-null mice had significantly lower levels of serum leptin under either a ND (2.720±1.732 pg/ml vs 1.419±1.023 pg/ml, **Table 1**, P<0.05) or a HFD feeding condition (13.157±8.824 pg/ml vs 4.52±5.588 ng/ml, P<0.001). Levels of serum leptin under fasting condition were also significantly reduced in *Fsp27* **^−/−^** *m*ice (**Fig. 2F**, **Table 1**). Consequently, food intake, which is regulated by plasma levels of leptin, was 10% higher in *Fsp27*-null mice compared to that of wildtype mice (**Fig. 2G**, 4.2±0.036 g/d vs 3.7±0.026 g/d, P<0.01). We also measured the levels of plasma TAG and NEFA and found that when animals were fed with a HFD, levels of plasma TAG were significantly lower in *Fsp27*-null mice under fasting conditions (**Table 1**, P<0.001). Levels of plasma non-esterified fatty acids (NEFA) were also significantly lower in *Fsp27*-null mice under both HFD and ND feeding or fasting conditions (**Table 1**)**.** Surprisingly, we observed lower levels of plasma ketone body under both ND and HFD fed conditions and under ND fasting condition for *Fsp27* **^−/−^** mice compared with that of wild type mice (**Table 1**). Levels of free cholesterol, LDL and HDL were similar between wild type and Fsp27 null mice (**Table 1**). Levels of plasma NEFA and TAG in *ob/ob*/*Fsp27* ^−/−^ mice were also lower than that of *ob/ob* mice (**Table 2**). Levels of plasma glucose under both fed and fasting conditions were similar between wild type and *Fsp27* ^−/−^ mice. However, levels of plasma insulin under ND and fasting condition were varied and generally higher in *Fsp27* **^−/−^** mice than that of wild type (**Table 1**). The rate of oxygen consumption measured by indirect calorimetry was approximately 20% higher in *Fsp27-*null mice than in wildtype mice under both ND and HFD conditions (**Fig. 2H**), suggesting of an increased whole-body energy expenditure in *Fsp27* **^−/−^** mice. As Fsp27 has been suggested to play a role in TAG storage and lipolysis [31], we measured the rate of lipolysis in WAT in the absence or presence of β-agonist (isoproterenol) and observed that WAT of *Fsp27* null mice had higher rate of basal lipolysis. The rate of β-agonist induced lipolysis was similar between wild type and *Fsp27* null mice (**Fig. 2I**, **Fig. S1C**). We then measured the body temperature at ambient conditions under both feeding and fasting conditions and observed that *Fsp27* ^−/−^ mice had slightly higher body temperature compared with that of wild type mice (**Fig. S2).** Upon exposure to cold, the core body temperature of wild type and *Fsp27* **^−/−^** mice remain similar at the first 30 min after cold exposure **(** **Fig. 2J**). However, the core body temperature decreased rapidly in *Fsp27* **^−/−^** mice after prolonged cold exposure (**Fig. 2J**). Our data therefore clearly suggest that *Fsp27-*deficiency in both wild type and *leptin* deficient mice could lead to a lean phenotype and less fat accumulation in the WAT.

### Improved insulin sensitivity in *Fsp27* ^−/−^ and *leptin*/*Fsp27* double deficient mice

**Figure 3:**
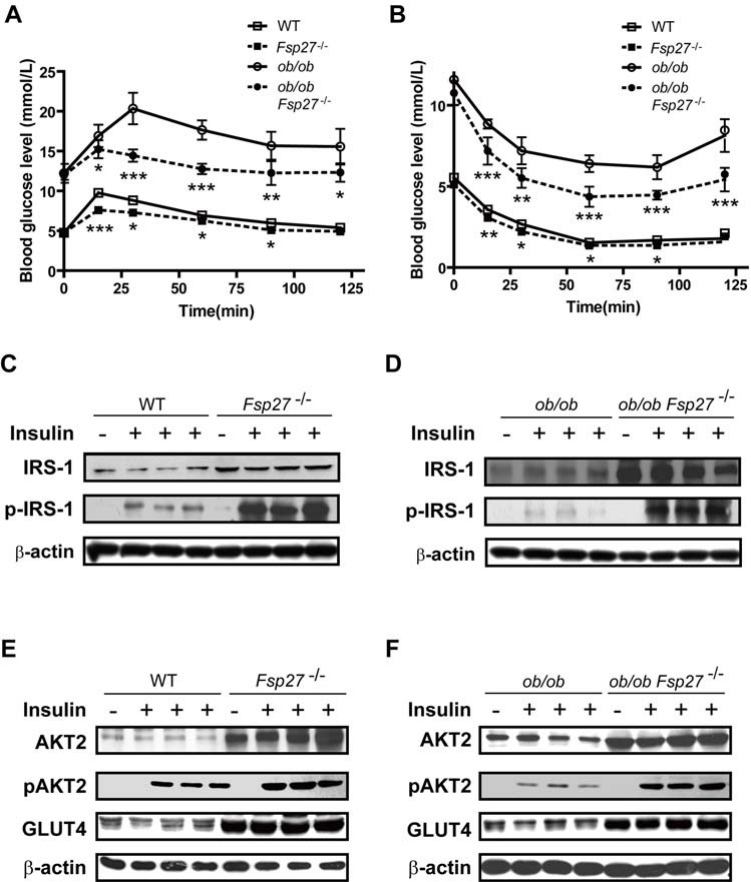
Increased insulin sensitivity in *Fsp27* ^−/^ and *ob/ob*/*Fsp27* ^−/−^ mice. GTT (A) and ITT (B) experiments using 10-week-old wild type (WT), *Fsp27* mutant (*Fsp27* ^−/−^), *leptin* deficient (*ob/ob*) and *leptin*/*Fsp27* double deficient (*ob/ob/Fsp27^−/−^)* mice. 6 mice from each group were used for the experiment. *p<0.05; **p<0.01; ***p<0.001. Western blot analysis for levels of IRS-1 and insulin stimulated IRS-1 tyrosine phosphorylation (p-IRS) in the WAT of wild type (WT) and Fsp27 mutant (Fsp27^−/−^) mice (C) and in the WAT of *leptin* deficient (*ob/ob*) and *leptin*/*Fsp27* double deficient (*ob/ob/Fsp27^−/−^)* mice (D). . E & F. Western blot analysis of total AKT2, phosphor-AKT2 (pAKT2) and GLUT4 protein levels in WAT of wild type (WT), *Fsp27* mutant (Fsp27^−/−^), *leptin* deficient (*ob/ob*) and *leptin*/*Fsp27* double deficient (*ob/ob/Fsp27^−/−^)* mice. β-actin was used as loading control.

The lean phenotype and lower plasma TAG and NEFA in *Fsp27* ^−/−^ and *ob/ob*/*Fsp27* ^−/−^ mice raised the possibility that their insulin sensitivity might be increased. We then measured the rates of glucose disposal and insulin sensitivity in *Fsp27* ^−/−^ and *ob/ob*/*Fsp27* ^−/−^ mice by glucose tolerance test (GTT) and insulin tolerance test (ITT). *Fsp27* ^−/−^ mice had significantly lower levels of blood glucose after administration of an exogenous load of glucose compared with that of wildtype mice, suggesting an enhanced glucose disposal (**Fig. 3A**). Whereas *ob/ob* mice had much higher levels of plasma glucose in the GTT test, a typical phenotype reflecting insulin resistance in these mice. Importantly, we also observed significantly reduced plasma glucose levels for *ob/ob*/*Fsp27* ^−/−^ mice (**Fig. 3A**) compared with that of *ob/ob* mice. These data suggest that mice with *Fsp27* deficiency in both wild type and *leptin* deficient background all have improved glucose disposal rate. When administered with excessive amounts of insulin in an ITT test, *Fsp27* ^−/−^ mice showed reduced levels of blood glucose compared with that of wildtype mice (**Fig. 3B, **P<0.001). In addition, *ob/ob*/*Fsp27* ^−/−^ mice had significantly lower levels of blood glucose compared with that of *ob/ob* mice in an ITT test (**Fig. 3B**). Improved glucose disposal and lower blood glucose levels in ITT experiments suggest that *Fsp27* ^−/−^ and *ob/ob*/*Fsp27* ^−/−^ mice have enhanced insulin sensitivity**.**

To gain mechanistic insight into the enhanced insulin sensitivity, we measured levels of IRS1, AKT2 and GLUT4 (principal mediators of insulin signaling pathway in adipocytes) and assessed the phosphorylation levels of IRS1 and AKT2 in the absence or presence of insulin. Levels of IRS1 were slightly increased in the WAT of *Fsp27* ^−/−^ mice (P<0.01, **Fig. 3C** **, Fig. S3A**). However, levels of tyrosine phosphorylation on IRS1 were drastically increased in the WAT of *Fsp27* mutant mice (approximately 8 fold higher, P<0.001, **Fig. 3C**, **Supplementary 3A**). In addition, levels of IRS1 tyrosine phosphorylation in the WAT of *ob/ob*/*Fsp27* ^−/−^ mice were also significantly increased compared with that in *ob/ob* mice (**Fig. 3D**, P<0.001, **Fig. S3A**). Levels of AKT2 were also significantly increased in *Fsp27* ^−/−^ and *ob/ob*/*Fsp27* ^−/−^ mice (**Fig. 3** **E&F**, P<0.01 & P<0.001, **Fig. S3B**). Furthermore, levels of phosphorylated-AKT2 in the presence of insulin were significantly increased in the WAT of *Fsp27* ^−/−^ and *ob/ob*/*Fsp27* ^−/−^ mice. No difference in IRS2 phosphorylation was observed in *Fsp27*-null mice (data not shown). Levels of IRS1 and AKT2, and their phosphorylated products in BAT, liver and skeletal muscle are similar between wild type and *Fsp27* ^−/−^ mice (**Fig. S4**), suggesting no change in insulin sensitivity in these tissues. Surprisingly, we observed significantly increased levels of GLUT4 in the WAT of *Fsp27* ^−/−^ and *ob/ob*/*Fsp27* ^−/−^ mice (**Fig. 3** **E&F**, **P<0.001,** **Fig. S3C).** These data suggest that insulin sensitivity in the WAT of *Fsp27* ^−/−^ and *ob/ob*/*Fsp27* ^−/−^ mice was improved possibly due to the increased protein levels of IRS1, AKT2 and GLUT4, as well as IRS1 tyrosine phosphorylation.

### Increases in mitochondrial size, activity and proteins in WAT of *Fsp27* ^−/−^ mice

**Figure 4:**
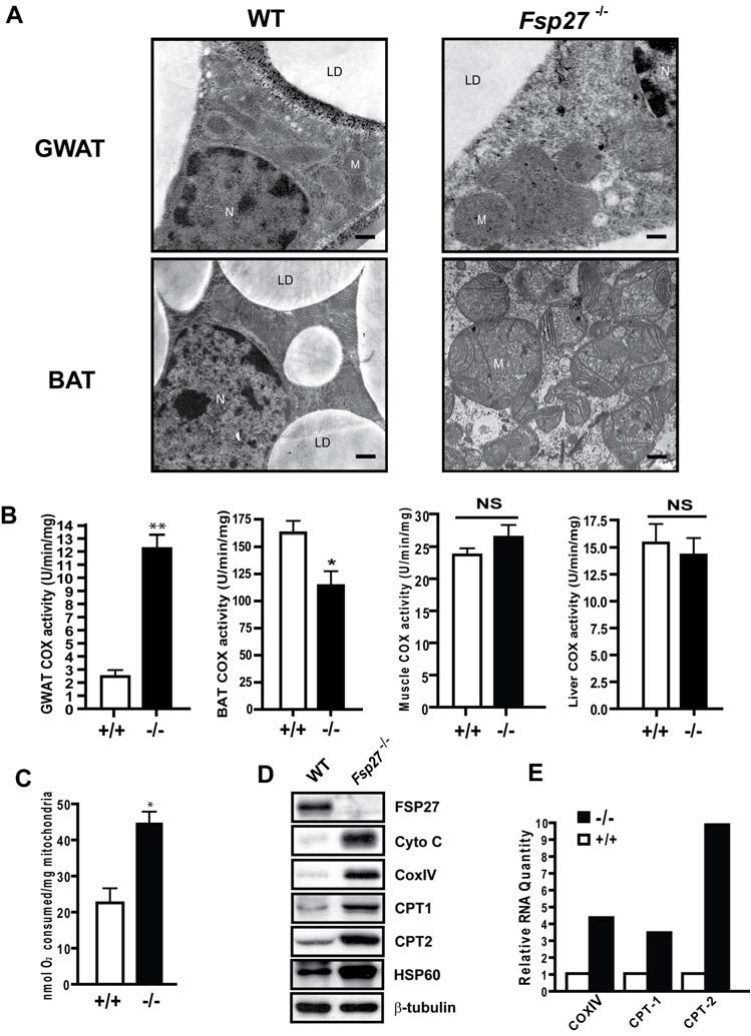
Increased mitochondria activity in the WAT of Fsp27 mutant mice. A. Electron microscope image of mitochondria in gonadal WAT (GWAT) (Scale Bar = 0.2 µm) and BAT (Scale Bar = 0.5 um) of wild-type and *Fsp27^−/−^* mice showing enlarged mitochondria in *Fsp27* ^−/−^ mice. N: nucleus, L: lipid droplet, M: mitochondria. B. Cytochrome oxidase IV (Cox) activity in GWAT, BAT, muscle and liver (n = 4). COXIV activity in *Fsp27* ^−/−^ mice is higher in GWAT (*p<0.05), but lower in BAT (**p<0.01) as compared with that of wild type mice. No difference in COX activity is detected in muscle or liver. C. Mitochondrial oxygen consumption rate is higher in WAT of *Fsp27* ^−/−^ mice. (*p<0.05) (n = 3). D. Western blot analysis of various mitochondrial proteins in the WAT of wild-type and Fsp27 mutant mice, showing higher level in Fsp27 mutant WAT. (n = 4), CytoC, cytochrome C. β-tubulin was used as loading control. D. Relative mRNA levels of COXIV, CPT1 and CPT2 in the WAT of wild type (+/+) and *Fsp27* mutant mice (−/−).

To investigate the underlying mechanism for reduced lipid accumulation, increased whole body metabolism in WAT and lean phenotypes in *Fsp27* null mice, we examined the mitochondrial morphology of *Fsp27* **^−/−^** white adipocytes by EM analysis. The mitochondria in wildtype white adipocytes were smaller and more compact. However, the mitochondrial volume appears to be dramatically increased in gonadal white adipocytes of *Fsp27*-null mice compared with that of wild type mice (**Fig. 4A**). Mitochondria in *Fsp27* **^−/−^** white adipocytes were full of straight or slightly wavy cristae that transverse the width of the mitochondria, a typical characteristic of metabolically active mitochondria (**Fig. 4A**). While brown adipocytes of wild type mice have larger mitochondria in their cytosol with dense and regular cristea aligned along the mitochondrial matrix, mitochondria from *Fsp27* **^−/−^** brown adipocytes were irregular in size and had significantly fewer cristea in its matrix, suggesting their lower activity in BAT (**Fig. 4A**). No change in mitochondrial morphology was observed in muscle cells and hepatocytes of wild type and *Fsp27* **^−/−^** mice (data not shown).

To investigate whether the increased mitochondrial size is correlated with enhanced activity, we measured the activity of mitochondrial cytochrome C oxidase (COX), which is the terminal electron acceptor that transfers electron from cytochrome C to molecular oxygen. We observed that COX activity in the mitochondria of *Fsp27* **^−/−^** WAT (12.29±0.7381 U/min/mg) is approximately 5 fold higher than that of wild type WAT (3.394±0.9319 U/min/mg, P≤0.01, **Fig. 4B**). In contrast, COX activity in BAT of *Fsp27* **^−/−^** mice (114.7±9.402 U/min/mg) was slightly lower than that of wild type mice (159±8.622 U/min/mg). Consistent with their decreased mitochondrial activity, levels of mitochondrial proteins such as Cpt1, Cpt2 and BAT specific proteins such as Ucp1 and PGC1 were decreased in the BAT of *Fsp27* **^−/−^** mice (**Fig. S5**). No difference in COX activity was observed in muscle and liver between wild type and *Fsp27* **^−/−^** mice (**Fig. 4B**). To further confirm that mitochondrial activity is increased in mutant WAT, we measured the rate of mitochondrial oxygen consumption from WAT of wildtype and *Fsp27* **^−/−^** mice using a Clark-type electrode. Mitochondrial oxygen consumption rate of *Fsp27* **^−/−^** WAT was 2 fold higher (44.52±3.186 mol/mg) than that of wild-type mice (22.39±4.037 mol/ng, P<0.05, **Fig. 4C** **).** These data all indicate that *Fsp27* **^−/−^** white adipocytes showed increased mitochondrial size and enhanced mitochondrial activity.

To assess the molecular basis of increased mitochondria activity in *Fsp27* **^−/−^** mice, we measured the expression levels of several genes, including cytochrome c, COXIV, CPT1 and CPT2, that are involved in mitochondrial oxidative phosphorylation and fatty acid oxidation. We observed that their protein levels were markedly increased in *Fsp27* **^−/−^** WAT. Hsp60, a chaperon protein localized in the mitochondrial matrix, was also significantly increased (**Fig. 4D**), consistent with the increased mitochondrial biogenesis in *Fsp27* **^−/−^** white adipocytes. We further measured their mRNA levels by semi-quantitative PCR and observed that the mRNA levels of COXIV, CPT1 and CPT2 were significantly increased in *Fsp27* **^−/−^** mice, consistent with the increase in their protein levels (**Fig. 4E**). These data suggest that WAT of *Fsp27* **^−/−^** mice have increased mitochondrial activity due to increases mitochondrial size and higher levels of mitochondrial gene expression.

### Up-regulation of crucial transcriptional factors and BAT-specific proteins in *Fsp27* ^−/−^ WAT

**Figure 5:**
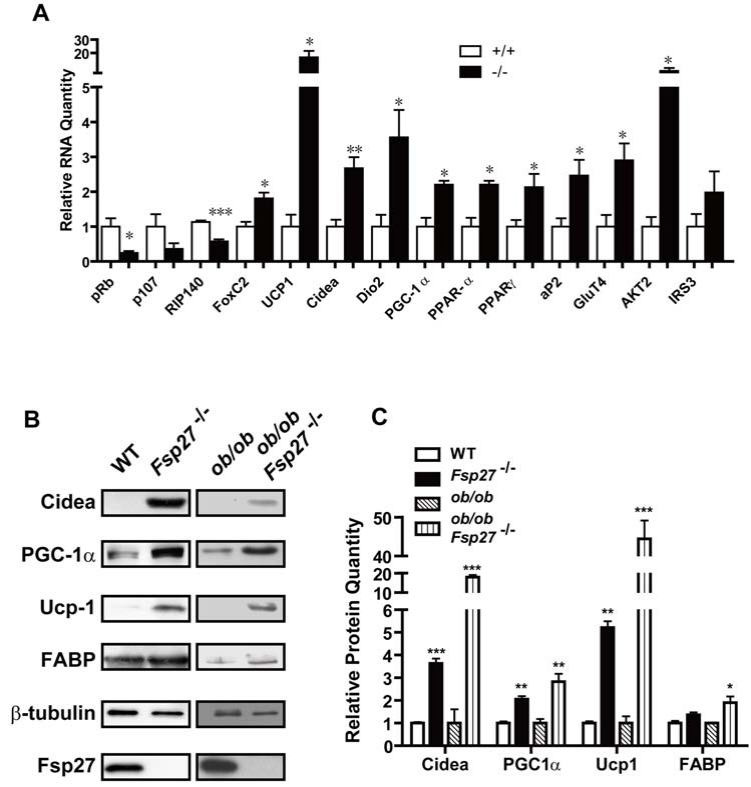
Altered expression levels of crucial metabolic regulators and BAT-specific factors in *Fsp27* mutant mice. A. Real-time PCR analysis of regulatory factors and BAT specific gene expression in the WAT of wild type (+/+) and *Fsp27^−/−^* mice (−/−). Significantly higher levels of BAT markers expression in Fsp27 mutant mice WAT were detected (*p<0.05; **p<0.01). B. Western blot analysis showing increased protein levels of Cidea, PGC1α, Ucp1 and FABP in the WAT of wildtype, *ob/ob*, *Fsp27^−^* ^/−^ and *ob/ob*/*Fsp27^−^* ^/−^of Fsp27 mutant mice. β-tubulin was used as loading control. C. Densitometric reading of relative levels of the indicated proteins in western blot analysis (as in B) were quantitated in these mouse strains. Similar experiments were done five times and the intensity of individual band in each western blot was quantified by TOTAL-LAB software (Nonlinear Dynamics, UK) and used for statistical analysis. The relative protein level in wildtype and ob/ob mice was designated as 1.0. **p<0.01.

The morphological resemblance of WAT to that of BAT, and the increased mitochondrial activity in WAT of *Fsp27* **^−/−^** mice, suggest that *Fsp27* **^−/−^** WAT might have acquired BAT-like properties. To determine the extent of shifting of the physiology and gene expression profiles of WAT to BAT in *Fsp27* **^−/−^** mice, we checked the expression levels of BAT-specific genes and several transcriptional regulators by real-time PCR analysis. We observed that the mRNA levels of the brown fat specific gene Ucp1 were significantly elevated in the WAT of *Fsp27* **^−/−^** mice compared with that of wild type mice **(approximately 20 folds, P<0.05,** **Fig. 5A** **)**. The mRNA levels of Cidea and Dio2, a protein that catalyzes the conversion of T4 (thyroxine) into the active substance T3 (3, 5, 3′-tri-iodothyronine) in BAT, were also significantly increased in the WAT of *Fsp27* **^−/−^** mice. In addition, mRNA levels of PGC1α, PPARα and PPARγ were all increased in the WAT of *Fsp27* ^−/−^ mice. Consistently, mRNA levels for FABP (aP2), a downstream target of PPARγ, were also increased (**Fig. 5A**). Importantly, FoxC2, a crucial regulator of cAMP pathway that promotes BAT differentiation, was increased in the WAT of *Fsp27* **^−/−^** mice. On the other hand, Rb, p107 and RIP140, factors that were negatively associated with WAT to BAT transformation were substantially decreased in WAT of *Fsp27* **^−/−^** mice (**Fig. 5A**). Furthermore, mRNA levels for GLUT4 and AKT2 were significantly increased (**Fig. 5A**), consistent with increased insulin sensitivity in *Fsp27* null mice. In accordance with their increased mRNA levels, protein levels for Cidea, PGC1α, Ucp1 and Fabp were significantly increased in WAT of *Fsp27* **^−/−^** mice (**Fig. 5B &C**). We then checked the protein levels of Cidea, PGC1α, Ucp1 and FABP in the WAT of *ob/ob* and *ob/ob/Fsp27* ^−/−^ mice and observed that their levels were all significantly up-regulated in *ob/ob*/*Fsp27* ^−/−^ mice (**Fig. 5B & C**). These data suggest that *Fsp27* acts upstream to control insulin signaling pathway and modulates programs that maintain WAT identity such as FoxC2, Rb, p107 and RIP140. Furthermore, Fsp27 regulates the expression of PPAR and PGC1 that in turn regulate their downstream targets such as Ucp1, Dio2 and Cidea expression.

### Differentiated *Fsp27* ^−/−^ MEFs display characteristics of brown adipocyte

**Figure 6:**
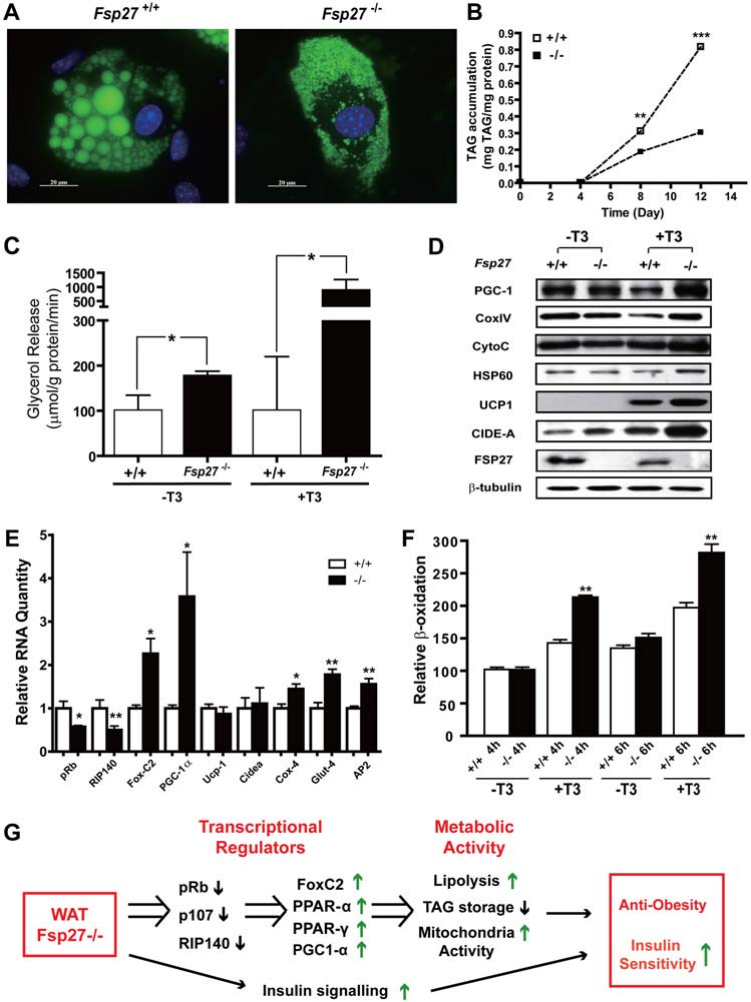
Differentiated Fsp27^−/−^ MEFs display many brown adipocyte characteristics. A. Smaller lipid droplets in differentiated *Fsp27* mutant mouse embryonic fibroblasts (MEFs). Lipid droplets were stained with BODIPY493/503, and nuclei were stained with Hochest 366243. Fields shown were visualized under fluorescence microscope at appropriate wavelengths for GFP (green) and Hoechst (blue). B. Lower TAG accumulation in differentiated *Fsp27^−/−^* MEFs (−/−) than in wildtype (+/+) cells. (n = 4) (**p<0.01; ***p<0.001). C. Increased basal lipolysis rate in differentiated Fsp27^−/−^ MEFs in the absence or presence of T3 (10 nM) in the differentiation medium. (n = 3) (*p<0.05; **p<0.01). D. Western blot analysis showing levels of various proteins in differentiated MEF cells in the absence or presence of T3 (10 nM). β-tubulin was used as a loading control. E. Relative mRNA levels of various genes in differentiated wildtype (+/+) and *Fsp27^−/−^*(−/−)MEFs. mRNA levels for Rb were measured using MEFs that were differentiated for 3 days. (n = 4) (*p<0.05). F. Higher levels of fatty acid β-oxidation rate in differentiated *Fsp27^−/−^* (−/−) MEFs as compared to wildtype (+/+) MEFs in the presence of T3 (10nM). (n = 4) (**p<0.01). Results of relative amount of ^3^H_2_O released into the medium after four (4h) or six (6h) hours chase of pre-labelled MEFs with cold palmitate. Wildtype oxidation rate at 4h is set to 100. G. Regulatory and metabolic pathways that are altered in the WAT of *Fsp27^−/−^* mutant mice, leading to anti-obesity and insulin sensitive phenotype.

To check whether the conversion of WAT to BAT in *Fsp27* **^−/−^** mice is cell autonomous or requires exogenous signals, we isolated mouse embryonic fibroblasts (MEFs) from wild type and *Fsp27* **^−/−^** mice and induced them to differentiate into adipocytes *in vitro*. The morphologies of differentiated MEFs from wildtype and *Fsp27* ^−/−^ mice are very different. Wildtype adipocytes have fewer but larger lipid droplets while differentiated *Fsp27* **^−/−^** MEFs possesses more but smaller lipid droplets (**Fig. 6A**). The small lipid droplets in *Fsp27* ^−/−^ adipocytes tend to cluster together, fully resembling the morphology seen in WAT from *Fsp27* **^−/−^** mice. Furthermore, levels of TAG from differentiated *Fsp27* **^−/−^** MEFs were significantly lower than that of differentiated wild type MEFs (**Fig. 6B**). The lower amount of TAG and smaller lipid droplets in *Fsp27* **^−/−^** adipocytes was not due to their defect in differentiation as the levels of adipocyte-specific markers such as *Fabp* and *Perilipin-A* were in fact slightly higher in *Fsp27* **^−/−^** adipocytes **(data not shown**). Furthermore, the lower amount of TAG in differentiated *Fsp27* **^−/−^** MEFs was not due to a decrease in FFA uptake as the rate of fatty acid uptake was similar between differentiated wildtype and *Fsp27* **^−/−^** MEFs (**data not shown)**. Consistent with data observed with WAT of *Fsp27* **^−/−^** mice, rates of basal lipolysis were all significantly increased in differentiated *Fsp27* ^−/−^ MEFs compared with that of wild type cells (**Fig. 6C, P** **<0.05)**. These data suggest that *Fsp27* deficiency in differentiated MEFs leads to lower TAG accumulation, increased basal lipolysis and smaller lipid droplets.

We then checked the expression levels of PGC1α, Ucp1, Cidea and mitochondrial proteins in differentiated *Fsp27* **^−/−^** MEFs in the presence or absence of T3. In the absence of T3, protein levels for PGC1α were similar for differentiated wild type and *Fsp27* ^−/−^ MEFs (**Fig. 6D**), which is in drastic contrast to the phenotypic changes seen between the wildtype and mutant adipocytes derive from the mice. In addition, protein levels for mitochondrial proteins for oxidative phosphorylation such as cytochrome-C and COXIV were also similar in differentiated wild type and *Fsp27* ^−/−^ MEFs. Furthermore, no up-regulation of Ucp1 and Cidea was observed in differentiated *Fsp27* ^−/−^ MEFs compared to wild type **(** **Fig. 6D** **)**. These data suggest that *in vitro* molecular induction of Ucp1 expression and upregulation of mitochondrial proteins for oxidative phosphorylation in *Fsp27* ^−/−^ adipocytes requires exogenous signals or factors, and are likely influenced by endocrine regulation. We then tested several factors known to promote BAT differentiation such as β-agonist, retinoid acid and T3 during MEF differentiation. We observed that in the presence of T3 in the differentiation medium, basal lipolysis rate (**Fig**. 6C) and protein levels for PGC1α were significantly increased in differentiated *Fsp27* ^−/−^ MEFs compared with that of wild type cells (**Fig. 6D**, **Fig. S6**, P<0.05). Ucp1 protein was also induced in the presence of T3 in differentiated wild type MEFs but its levels were increased by approximately two fold in differentiated *Fsp27* ^−/−^ MEFs (P<0.05). Levels of Cidea were also significantly increased by more than 3 fold in differentiated *Fsp27* ^−/−^ MEFs **(** **Fig. 6D** **, Fig. S6,** P<0.05) in the presence of T3, although no difference in mRNA levels for Ucp1 or Cidea was observed between wild type and *Fsp27* ^−/−^ MEFs. Furthermore, levels of mitochondrial proteins such as COXIV, cytochrome C and Hsp60 were also higher. Consistent with the increase in PGC1α protein levels, the mRNA levels for PGC1α were also significantly increased (approximately 4 fold) and FoxC2 (2 fold) in differentiated *Fsp27* ^−/−^ MEFs in the presence of T3 **(** **Fig. 6E** **)**. Consistent with our *in vivo* data, levels of Rb and RIP140 mRNAs were significantly decreased in differentiated *Fsp27* ^−/−^ MEFs. The mRNA levels for GLUT4 were also increased in differentiated *Fsp27* ^−/−^ MEFs. The rate of fatty acid oxidation, an indicator of mitochondrial activity, is increased in differentiated *Fsp27* ^−/−^ adipocytes in the presence of T3 at various time points tested **(** **Fig. 6F** **)**. These data suggest that *Fsp27* ^−/−^ MEFs attain certain characteristics of brown adipocyte-like cells *in vitro* and that the thyroid hormone T3 plays a critical role in this phenotypic changes.

## Discussion

Through genetic deletion of *Fsp27*, we have found that *Fsp27* deficiency causes reduced white fat pads, decreased adiposity, increased whole body metabolism, enhanced insulin sensitivity and a lean phenotype not only in a wild type background but also in *leptin* deficient obese mice. The anti-obesity effect of *Fsp27* deficiency in wild type and *ob/ob* background is likely due to increased energy expenditure in WAT of *Fsp27* ^−/−^ mice as it has markedly increased mitochondrial activity, which could in turn lead to the increased consumption of TAG and FFA in WAT and enhanced overall whole body energy expenditure. Furthermore, we could rule out the contribution of other tissues such as muscle and liver to the decreased overall adiposity as we observed no difference in morphology and mitochondrial activity in these tissues between wild type and *Fsp27* ^−/−^ mice.

Besides its role in regulating adiposity, Fsp27 also plays an important role in modulating insulin sensitivity. Both *Fsp27* ^−/−^ and *ob/ob/Fsp27* ^−/−^ mice have increased glucose uptake and improved insulin sensitivity. The mechanism by which *Fsp27* ^−/−^ mice had increased insulin sensitivity is likely due to an increased expression of GLUT4 and AKT2 and increased tyrosine phosphorylation of IRS1 in the WAT. We only notice a marginal difference in glucose levels after glucose and insulin administration under normal diet condition (**Fig.** **3A & B**), which is in contrast to the drastic difference in the phosphorylation of IRS1 and AKT2 (**Fig.** **3C to 3F**) in the WAT of *Fsp27* ^−/^ mice. Glucose uptake and insulin sensitivity are contributed by multiple organs such as adipose tissue, liver and muscle, depending on the status of energy homeostasis within the body. As we observed no difference in the levels of AKT phosphorylation in the skeletal muscle, liver or BAT, it is possible that under normal diet feeding condition when animals are insulin sensitive, the impact of increased insulin sensitivity in WAT on whole body glucose level control would be marginal. However, under the condition that animals are already insulin resistant (such as in the *ob/ob* background), increased insulin sensitivity in WAT of *Fsp27* deficiency in *leptin* and *Fsp27* double deficient mice caused drastically reduced glucose levels after glucose and insulin administration.

We observed that unlike wild type white adipocytes that contain a large unilocular lipid droplet, *Fsp27* ^−/−^ white adipocytes have small but numerous lipid droplets in their cytosol, with nuclei localize at the center of the cell. Furthermore, *Fsp27* ^−/−^ white adipocytes contain significantly larger mitochondria, dramatically higher mitochondrial activity and markedly elevated levels of many mitochondrial proteins for fatty acid oxidation, electron transport chain and TCA cycles. BAT enriched proteins such as Ucp1, Cidea and Dio2 were all significantly up-regulated in *Fsp27* ^−/−^ white adipocytes. Expression of PPAR family proteins (PPARα and γ) and PGC1α, crucial factors for controlling adipocytes differentiation, lipolysis, fatty acid transport and oxidation, glycolysis and uncoupling are also upregulated in WAT of *Fsp27* ^−/−^ mice. Furthermore, the increase in Dio2 expression could also boost the local concentration of T3, and serve as a source for systemic T3 in WAT of *Fsp27* ^−/−^ mice, resulting in the increased PGC1α and Ucp1 expression. These alterations all point to a possibility that the WAT tissue of *Fsp27* ^−/−^ mice has acquired certain BAT-like properties, and hence was transformed from a typical energy storage tissue into an energy consuming tissue. Although the exact mechanism by which *Fsp27* ^−/−^ WAT is transformed into a BAT-like tissue is not clear, we observed enhanced expression of genes regulating cAMP signaling pathway such as FoxC2 in WAT of *Fsp27* ^−/−^ mice. Enhanced FoxC2 expression could contribute to the increased expression of PGC1α and Ucp1. We also observed dramatically reduced expression of Rb and its homolog p107 that were known to negatively regulate PGC1α expression and BAT differentiation. Furthermore, we observed reduced expression of a co-repressor of many nuclear receptors, RIP140, in the WAT of *Fsp27* ^−/−^ mice. Therefore, the attainment of BAT–like property in the WAT of *Fsp27* ^−/−^ mice is likely to be a combinatory effect of activation of pathways promoting the conversion and downregulation of pathways that inhibit the conversion of BAT to WAT. Fsp27 appears to act somewhere upstream to control signaling networks such as cAMP pathway, Rb, p107 and RIP140 expression and insulin signaling that maintains WAT identity (**Fig.** **6G**). The phenotype in *Fsp27* ^−/−^ WAT is reminiscent of that of FoxC2 overexpression transgenic mice [22] and p107 and Rb knockout mice [23], where PGC1α and Ucp1 are upregulated and that WAT of these genetically modified mice all showed brown adipocyte-like metabolic activity. However, these *Fsp27* ^−/−^ WAT may not fully function as a brown adipocyte as the relative levels of Ucp1 is only approximately 5–10% of that of wild type BAT (data not shown). The level of PGC1α and Cidea in *Fsp27* ^−/−^ WAT were also not as high as in wild type BAT. Similarly, mitochondrial cristea of *Fsp27* ^−/−^ white adipocytes were not as tightly packed and regularly arranged as that of BAT.

Remarkably, the BAT-like phenotype in the WAT of *Fsp27* ^−/−^ mice can be mostly reproduced in MEFs differentiated *in vitro*. Our observations suggest that the acquirement of BAT-like property in WAT may require two steps, the first of which is T3-independent and cell autonomous, and the subsequent step is T3-dependent. In the first step, the changes mainly include smaller lipid droplets, increased lipolysis, and reduced TAG storage in the basic differentiation medium that contains pioglitazone and insulin. In the second step, which requires T3 in the differentiation medium, fatty acid β-oxidation was increased, accompanied by the increased protein levels of brown fat specific genes (PGC1α, Cidea, Ucp1) and mitochondrial proteins such as Cox IV. In this study, we have also revealed the importance of T3 in increasing mitochondrial activity and altering transcriptional program in the WAT of *Fsp27* ^−/−^ mice. Fsp27 could functionally antagonize the T3 action, which is a pleiotropic hormone regulating the expression of many genes involved in adipocyte differentiation, lipogenesis and lipolysis, and controls the induction and maintenance of the WAT identity *in vivo*. Based on these data, the phenotype of reduced lipid droplet size, increased lipolysis and reduced TAG storage appears to be the primary defects in the WAT of *Fsp27* ^−/−^ mice, while increased mitochondrial activation and up-regulation of BAT specific markers are secondary effects. The mechanism by which Fsp27 controls lipid droplet function and mitochondrial activity is not clear. As certain portion of endogenous Fsp27 proteins are localized to the lipid droplet [31], [32] **(** **Fig. 1D** **)**, it may function to control lipid droplet formation or inhibit lipolysis. Increased mitochondrial oxidative activity and Ucp1 expression in the WAT of *Fsp27* ^−/−^ mice may be a result of increased release of fatty acids to the cytosolic fraction. Attainment of a BAT-like cell type with increased mitochondrial activity in *Fsp27* ^−/−^ white adipocytes may reflect a feedback control in regulating TG storage, resulting from a defect in lipid droplet formation, lower TG storage and a lean phenotype. Although *Fsp27* ^−/−^ and *Perilipin* (*Plin)* ^−/−^ mice have a similar lean phenotype, the molecular mechanism by which *Fsp27* and *Perilipin* control energy storage and lipid droplet formation appears to be different [31], [33], [34]. *Plin* ^−/−^ mice had a defect in lipid storage due to increase lipolysis. As a result, lipids were channelled into the circulation and other tissues. No increase in mitochondrial activity and upregulation of mitochondrial gene expression was observed in *Plin* ^−/−^ mice. Fsp27, on the other hand, appears to control lipid storage and insulin sensitivity by regulating the expression levels of FoxC2, Rb/p107, RIP140, PPAR, PGC1, AKT2, GluT4 and mitochondrial proteins in addition to regulating lipid droplet size.

Paradoxically, we observed larger lipid droplets, slightly reduced mitochondrial protein expression and mitochondrial activity, irregular structure of mitochondrial cristae and reduced levels of mitochondrial proteins in *Fsp27* ^−/−^ brown adipocytes. All these changes are somewhat opposite to the changes observed in the *Fsp27* ^−/−^ WAT and are apparently associated with BAT dysfunction. The precise role of Fsp27 in regulating BAT function is not clear. The reduced activity in the BAT of *Fsp27* ^−/−^ mice may be related to a decrease of sympathetic innervation and activity surrounding the BAT. Further analysis will be needed to address Fsp27's molecular role in BAT. Furthermore, increased WAT activity coupled with reduced BAT activity was also observed in several genetically modified lean mouse models such as transgenic mice over-expressing A-ZIP, a nuclear form of SREBP1, Ucp1 and FoxC2 in WAT [22], [35]–[37] as well as Caveolin-1 knockout mice[38]. It is possible that increased WAT activity could in turn inhibit BAT activity.

We observed that *Fsp27* ^−/−^ mice had slightly increased body temperatures under both feeding and fasting conditions in particular for female mice under high fat diet condition, indicating a possible increase in thermogenic activity. We found that the body temperature within the first hour of cold exposure was similar between wild type and *Fsp27* ^−/−^ mice. Intriguingly, the body temperature in *Fsp27* ^−/−^ mice became lower than the wild type mice at one hour of cold exposure and decreased even further thereafter. Therefore, *Fsp27* ^−/−^ mice appear to have slightly increased thermogenic activity in ambient condition, but they become hypothermic with prolonged cold exposure. The slight increase in thermogenic activity in ambient temperature in *Fsp27* ^−/−^ mice may be due to increased Ucp1 expression and enhanced mitochondrial activity in their WAT. However, we have evaluated Ucp1 protein levels in WAT of *Fsp27* ^−/−^ mice and observed that the amount of Ucp1 in *Fsp27* ^−/−^ WAT is approximately only 5% of that in wild type BAT (data not shown). Furthermore, mitochondrial cristea structure of *Fsp27* ^−/−^ white adipocytes was not arranged as regularly as wild type brown adipocytes. In addition, significantly lower amount of TG was stored in *Fsp27* ^−/−^ white adipocytes, resulting in limited fuel for WAT to produce heat when exposed to cold. Therefore, although the white adipocytes of *Fsp27* ^−/−^ mice had increased Ucp1 expression, their capacity in generating heat and to increase body temperature when animals were exposed to cold with prolonged time remains unclear. Importantly, maintenance of body temperature under prolonged cold treatment would require coordination between BAT (non-shivering) and muscle (shivering). As *Fsp27* ^−/−^ mice are leaner and had a problem storing lipid, hence hypothermia seen in *Fsp27* ^−/−^ mice may be due to the reduced lipid storage, which translates into lesser fuel for muscle activity. We speculate that the BAT-like property in the WAT of *Fsp27* ^−/−^ mice could contributes to the increased energy expenditure and lean phenotype, but could not serve to maintain body temperature when animals are exposed to prolong cold treatment. Taken together, our study has clearly demonstrated that *Fsp27* acts as a novel regulator to control gene expression of crucial metabolic regulators, mitochondrial activity and white adipocyte identity. Therefore, *Fsp27* could serve as a potential therapeutic target for the control of obesity and diabetes.

## Materials and Methods

### Generation of Fsp27 knockout mice

Procedures for the isolation of *Fsp27* genomic clones, generation of *Fsp27*-deficient mice, and routine maintenance of mouse strain were essentially the same as previously described [6]. Animals were fed with either normal chow diet (5053, PicoLab Rodent Diet 20) or high-fat diet (D12331, 58% of kilocalories from fat; Research Diet). Mice experiments were carried out in the *In Vivo* Modeling Unit, Institute of Molecular and Cell Biology and animal facility in Department of Biological Sciences and Biotechnology, Tsinghua University. Mice handling procedures were in accordance with the Responsible Care and Use of Laboratory Animals (RCULA) guideline set by the National University of Singapore and Tsinghua University.

### Genotyping, RNA extraction, semi-quantitative real-time PCR

Genomic DNA was isolated from mouse tails as previously described [39]. Total RNA was isolated using the TRIzol reagent (Invitrogen, USA). The first-strand cDNAs were synthesized from 1 µg of total RNA with oligo-(dT) 20 primers using Superscript III RT kit (Invitrogen, USA) according to the manufacturer's protocol. Real-time PCRs were performed with ABI SYBR GREEN PCR Master Mix in the MX3000P real-time PCR system (Stratagene, USA) according to the manufacturer's instruction. Results were normalized to β-actin level in each sample. Primer sequences for genotype and real-time PCR analyses are available upon request.

### Adiposity index, electron microscopy, histological analysis, whole body O_2_ consumption, food intake measurement and core body temperature

Procedures for adiposity index analysis, electron microscopy, H&E staining and whole body O**_2_** consumption were essentially the same as described by Li et al [26]. For histological analysis, WAT was fixed, dehydrated, embedded and transversely sectioned into 5 µm piece for further staining. For whole body O_2_ consumption assessment, mice were adapted for 8 hours in the metabolic chamber before reading were taken periodically at 15 min intervals for the next 10 h. Mice were given free access to food and water during measurement. For food intake measurement, two or three mice were house in a cage. The amount of normal diet consumed was carefully monitored at 10:00 a.m. local time very morning for a period of one month. Measurement of core body temperature of the mice is essentially the same as describe by Zhuo et al [6]

### Glucose and insulin tolerance tests (GTT and ITT)

We carried out glucose and insulin tolerance tests (GTT and ITT) by intraperitoneal injection of glucose (1.0 g per kg of body weight) and recombinant human insulin (0.5 unit per kg body weight) into fasting mice (16 hour or 4 hour for GTT and ITT, respectively). We took tail blood samples before (0 min) and 15, 30, 60, 90, and 120 min after injection, to measure glucose levels. Glucose levels were measured by a blood glucose meter (Roche).

### Blood chemistry

NEFA, TG, HDL, LDL and Cholesterol were quantified using NEFA-C, L-Type Triglyceride H, HDL-Cholesterol E, L-Type LDL-C and Cholesterol E kits according to manufacturer's instructions (Wako Pure Chem, Japan). Insulin levels (CRYSTAL CHEM., USA) were measured according to manufacturer's instruction.

### Mitochondria O2 consumption from tissue

Mitochondria were isolated essentially as described [40]. Isolated mitochondria was resuspended in MRB buffer (120 mM KCl, 20 mM sucrose, 3 mM HPEPS-KOH, pH 7.2, 3 mM KH**_2_**PO**_4_**, 2 mM MgCl**_2_** and 2 mM EGTA) containing 0.5% (w/v) BSA. 200 µg of mitochondrial protein was added to a chamber equipped with a Clark-type O**_2_** electrode (Hansatech, USA) that contained 1 ml of MRB buffer maintained at 37°C. Mitochondrial activity measurement was initiated by the addition of 5 mM succinate and 1 mM ADP. Rate of oxygen consumption was monitored for 5-8 minutes before termination of experiment with 1mM KCN.

### Cytochrome oxidase activity

Cytochrome oxidase activity (Cox) activity was measured as described [21] with the following modifications. Reduced cytochrome c was prepared by incubating 5 mM DTT with 21.8 mM cytochrome C on ice for 15 min. To test Cox activity, tissue was homogenized in Buffer C (30 mM potassium phosphate, pH 7.4, with a protease inhibitor cocktail [Roche, USA]) and centrifuged at 600g for 2 minutes to remove tissue clumps and fat cake. 50 µg of total protein from the infranatant was added to Buffer C containing 0.01 µmol of reduced cytochrome C and measured immediately with a spectrophotometer. Cytochrome oxidase activity was monitored as the change of OD 550/565 ratio per unit time.

### Lipid extraction and TLC assay

Total lipid was isolated from mouse tissue or cells as previously described [41]. Dried lipids were reconstituted in chloroform/methanol 2∶1 and loaded onto a TLC plate (Sigma, USA). Lipids were resolved in hexane/diethyl ether/acetic acid 70∶30∶1 v/v. TLC plates were sprayed with 10% CuSO4 in 10% phosphoric acid and developed by drying in a 150°C oven.

### Western blot analysis

Tissue or cultured cells for western blot analysis of total lysate were lysed in RIPA buffer (20 mM HEPES-KOH pH 7.5, 150 mM NaCl, 1 mM EDTA, 10% Glycerol, 0.5% sodium deoxycholate, 1% NP40, 0.1% SDS and protease inhibitor) and analysed as previously described [6]. Western blot analysis for insulin stimulated phosphorylation of IRS1, AKT and GLUT4 was performed as described [26]. Fsp27 and Cidea was detected using polyclonal antibody raised against mouse Fsp27 and mouse Cidea by injecting His-Tagged truncated Fsp27 (aa 1–190) and Cidea (aa 1–195) into rabbit respectively as previously described [42]. Antibodies against anti-tubulin (Sigma), anti-rat FABP (Alpha Diagnostic.), anti-cytochrome c (Pharmingen), anti-CoxIV (Molecular Probes), anti-Cpt1 (Alpha Diag.), anti-Cpt2 (Alpha Diag.), anti-UCP3 (Research Diag. Inc.), Hsp60 (Chemicon), anti-Pgc1a (Chemicon), Ucp1 (Research Diag. Inc.), PGC1α (Chemicon), IRS-1 (Upstate), phosphotyrosine (Upstate), Akt (Cell Signaling), Phospho-Akt (Cell Sigaling), PPARα (Abcam), PPARδ (Abcam), PPARγ (Research Diag. Inc.) and β-tublin (Sigma) were used for western blot analysis.

### Immunofluoresence assay

Immunofluorescence study was carried out on cells grown on cover-slips. Cells were fixed in 3% paraformaldehyde/PBS solution for 30 min at room temperature, followed by permeabilization with 0.1% saponin/PBS for 15 mins. The cells were then incubated with purified Fsp27 antibody and perilipin antibody (Research Diag. Inc.). In MEF cells, intracellular lipids were visualize with 20 µg/ml Bodipy 493/503. Fluoresencing cells were visualize with Zeiss LSM510Meta cofocal and Zeiss Axiovert 200M microscope.

### Cell culture

3T3-L1 cells were maintained in Dulbecco's modified Eagle's medium (DMEM, Invitrogen, USA) containing 10% fetal bovine serum (FBS, Invitrogen, USA). Differentiation of 3T3-L1 cells were initiated at 2 days post-confluent cells by the addition of 5 µg/ml of insulin, 1 µM dexamethasone (DEX), and 0.5 mM isobutylmethylxanthine (IBMX) for 2 days and subsequently replaced by DMEM/FBS supplemented only with 5 µg/ml insulin for a further 2 days before replacing it with normal culture medium. Mouse embryonic fibroblasts (MEF) were isolated from 13.5 day wild-type and *Fsp27* mutant mouse embryos as described [43]. Passage 2 of MEF was used for differentiation. For the induction of MEF to differentiate into adipocytes, MEF cells were grown for 2 days post confluence, and then differentiated in DMEM/FBS supplemented with 8 µg/ml D-Pantothenic acid, 8 µg/ml Biotin, 1 µM DEX , 0.5 mM IBMX, 5 µg/ml insulin and 1.0 µM pioglitazone (TAYANG Pharmaceutical Industry Co. LTD, China), with or without 0.5 µM 3, 5, 3′-tri-iodothyronine (T3). Two days later, the medium was changed to DMEM /FBS, 8 µg/ml D-Pantothenic acid, 8 µg/ml Biotin, 5 µg/ml insulin, 1 µM pioglitazone, with or without 0.5 µM T3. Cells were collected after 8 days of differentiation and used for further experiments.

### Fatty acid β-oxidation

The procedure for measurement of fatty acid oxidation in MEF was essentially the same as described [44]. MEF cells were seeded in 12-well plates. After 8 days of differentiation, the cells were washed three times with HBSS (Hanks' balanced salt solution with calcium and magnesium, 10 mM HEPES) buffer and prelabeled with [9, 10(n)-^3^H] palmitatic acid (1.0 µCi/well) with 22 µM unlabeled palmitic acid and 0.5 mg/ml fatty acid-free BSA in HBSS. After incubation at 37°C for 2 h, cells were washed with HBSS three times and incubated in 0.5 ml HBSS buffer. At each time point, the reaction medium was transferred to glass tube and the cells were then washed 3 times with 0.5 ml HBSS which was collected in the same tube. The resultant medium was extracted with 8 ml Chloroform/Methanol (2∶1), and the aqueous phase was collected and used for radioactivity measurement in a liquid scintillation counter.

### Lipolysis assay in WAT and MEF cells

WAT isolated from three month old mice were cut into small pieces and 8 days post differentiated MEF were used for lipolysis assay [45]. Tissue pieces or cells were incubated in DMEM (without phenol red) containing 1% fatty acid free BSA with or without 1 µM isoproterenol as indicated. 100 µl of medium was withdrawn at the indicated time points and used for the assay. Glycerol level was determined using Free Glycerol determination kit according to the manufacturer's instruction (Sigma, USA).

### Statistical analysis

All statistical analyses were calculated using Graphpad Prism v4.0. Data consolidated were expressed as mean±sem and p value was calculated using non-parametric student t-test.

## Supporting Information

Figure S1 Generation of Fsp27 knockout mice. A. Fsp27 partial genomic structure, gene targeting construct, and expected homologous recombinant allele. Transcriptional disruption is achieved with a copy of the Neo gene being inserted in place of exon 2 and part of exon 3 in the opposite direction to transcriptional orientation. B. Total triacylglycerol content (TAG) in skeletal muscle (SM) and liver of 3 months old wildtype (+/+) and Fsp27−/− (−/−) mice (n = 3). C. Lipolysis rate of WAT from wildtype (+/+) and Fsp27−/− (−/−) mice treated with (Iso) or without (Basal) 1uM isoproterenol (n = 4) for the indicated time.
(0.77 MB TIF)

Figure S2 Body temperature of animals under ambient conditions. Retal temperature was taken from 6 months old wildtype (+/+) and Fsp27−/− (−/−) mice fed ad libitum with normal diet (ND) or high fat diet (HFD) or fasted for 18 hours (ND: Male: +/+: n = 16, −/−: n = 17; Female: +/+: n = 16, −/−: n = 16. HFD: Male: +/+: n = 18, −/−: n = 19; Female: +/+: n = 15, −/−: n = 22.).
(0.59 MB TIF)

Figure S3 Densitometric reading of relative protein level in western blot analysis performed for, A. IRS1 or phosphor-IRS1 (pIRS1), B. AKT2 or phosphor-AKT2 (pAKT2) and C. GLUT4 in WAT of 3 months old wildtype (WT), Fsp27−/−, leptin deficient (ob/ob) and leptin/Fsp27 double deficient (ob/ob/Fsp27−/−) mice (n = 3).
(0.32 MB TIF)

Figure S4 No difference of levels of AKT and phosphor-AKT in Fsp27−/−and ob/ob/Fsp27−/− mice. 3 months old mice that were fasted for 4 hours were intraperitoneally injected with 40mg/kg body weight of insulin. AKT proteins were immunoprecipitated with antibody against AKT and subsequently immunoblotted with antibodies again AKT or phosphor-AKT (pAKT). A, C& E. Western blot analysis for levels of total AKT and pAKT stimulated with and without insulin in BAT, skeleton muscle (SM) and liver of wild type (WT) and Fsp27 mutant (Fsp27−/−) mice. B, D& F. Western blot analysis for levels of total AKT and insulin stimulated pAKT in BAT, skeletal muscle (SM) and liver of ob/ob and ob/ob/Fsp27−/− mice. Actin was used as the loading control.
(1.80 MB TIF)

Figure S5 Western Blot analysis of total BAT tissue lysate from wildtype (Fsp27+/+) or Fsp27 null (Fsp27−/−) mice. β-tubulin was used as the loading control. Each panel is a representative of 4 individual experiments. Tubb represents tubulin.
(1.03 MB TIF)

Figure S6 Densitometric reading of relative protein level in western blot analysis performed for the indicated protein of wildtype (Fsp27+/+) or Fsp27 null (Fsp27−/−) mice in Day 8 post-differentiated MEF cells with (+T3) or without (−T3) triiodothyronine (n = 3).
(0.44 MB TIF)
