# Supplementary figures and images for: Up-Regulation of Mitochondrial Activity and Acquirement of Brown Adipose Tissue-Like Property in the White Adipose Tissue of Fsp27 Deficient Mice
Source: PLoS One. 2008 Aug 6;3(8):e2890. doi: 10.1371/journal.pone.0002890 (PMC2483355; doi:10.1371/journal.pone.0002890)

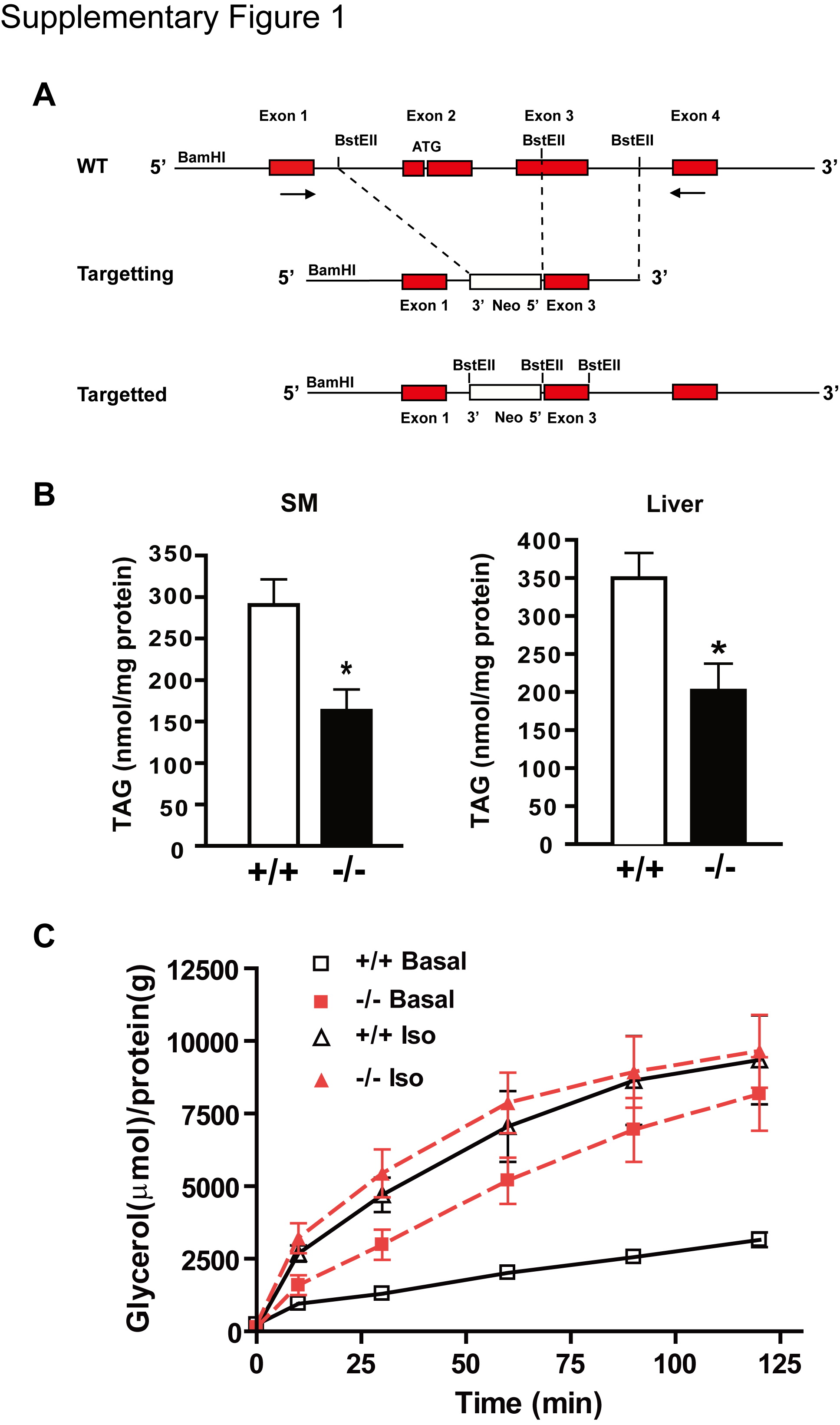

Supplement: Figure S1 — Generation of Fsp27 knockout mice. A. Fsp27 partial genomic structure, gene targeting construct, and expected homologous recombinant allele. Transcriptional disruption is achieved with a copy of the Neo gene being inserted in place of exon 2 and part of exon 3 in the opposite direction to transcriptional orientation. B. Total triacylglycerol content (TAG) in skeletal muscle (SM) and liver of 3 months old wildtype (+/+) and Fsp27−/− (−/−) mice (n = 3). C. Lipolysis rate of WAT from wildtype (+/+) and Fsp27−/− (−/−) mice treated with (Iso) or without (Basal) 1uM isoproterenol (n = 4) for the indicated time. (0.77 MB TIF) [file pone.0002890.s001.tif]

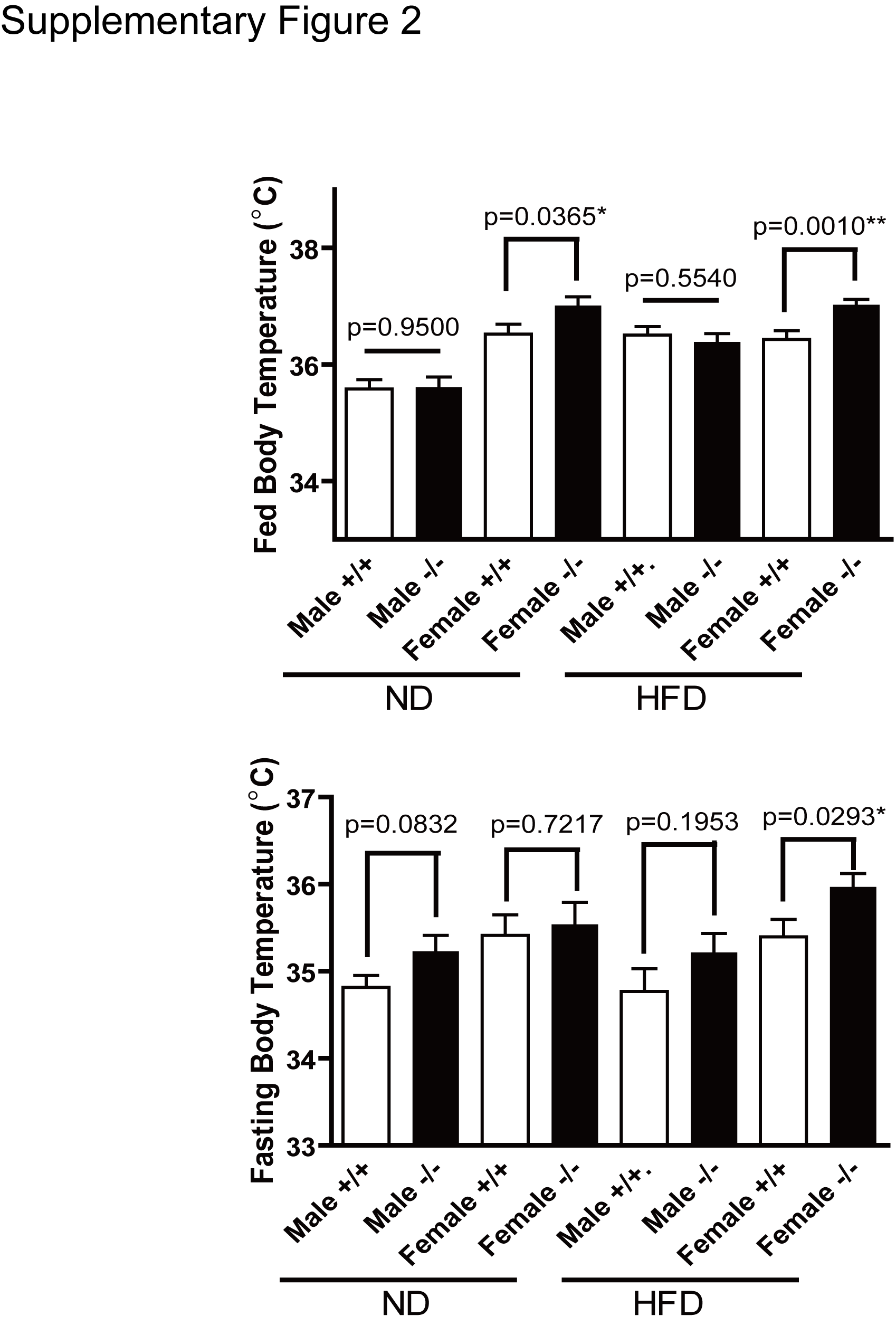

Supplement: Figure S2 — Body temperature of animals under ambient conditions. Retal temperature was taken from 6 months old wildtype (+/+) and Fsp27−/− (−/−) mice fed ad libitum with normal diet (ND) or high fat diet (HFD) or fasted for 18 hours (ND: Male: +/+: n = 16, −/−: n = 17; Female: +/+: n = 16, −/−: n = 16. HFD: Male: +/+: n = 18, −/−: n = 19; Female: +/+: n = 15, −/−: n = 22.). (0.59 MB TIF) [file pone.0002890.s002.tif]

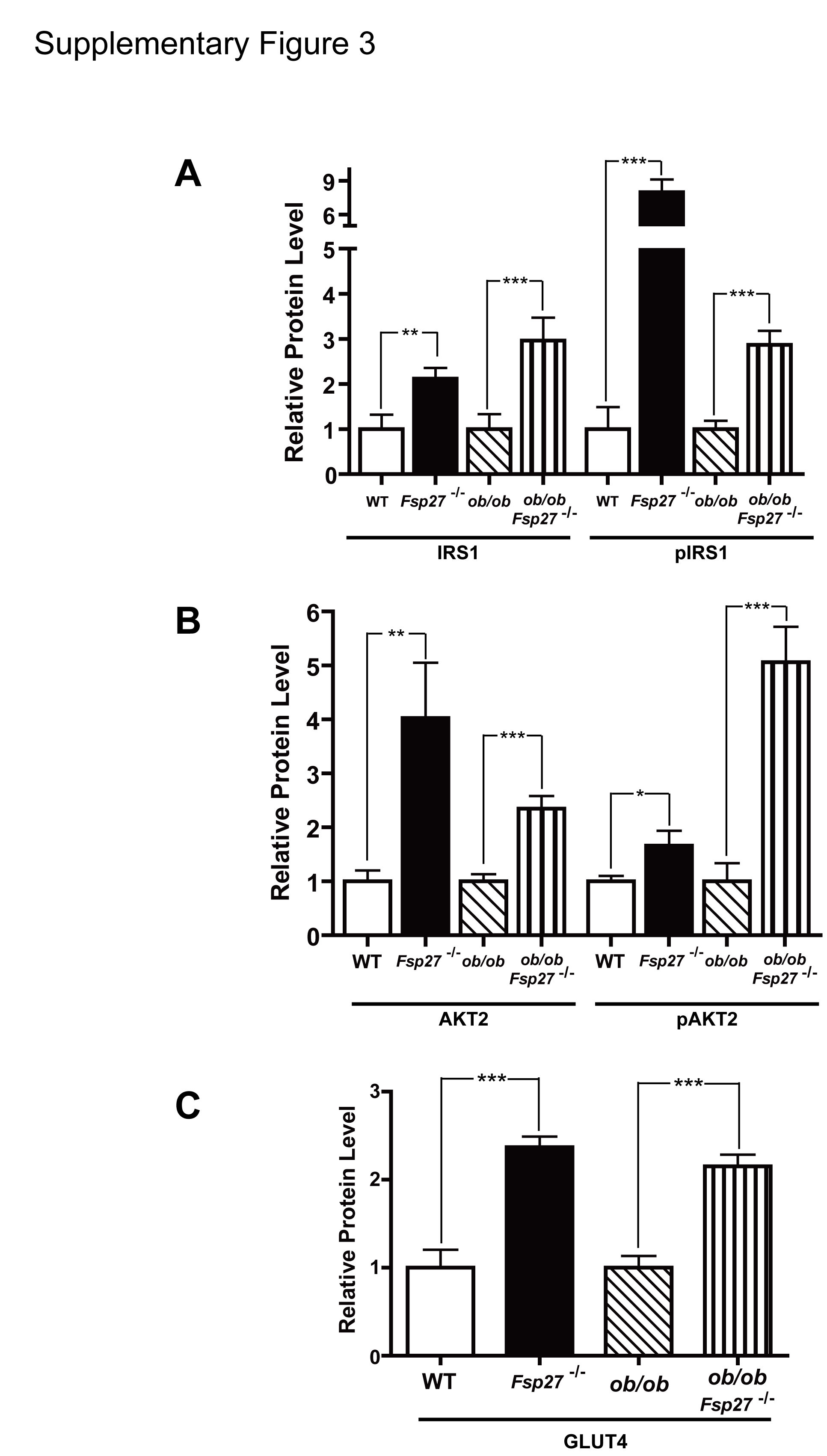

Supplement: Figure S3 — Densitometric reading of relative protein level in western blot analysis performed for, A. IRS1 or phosphor-IRS1 (pIRS1), B. AKT2 or phosphor-AKT2 (pAKT2) and C. GLUT4 in WAT of 3 months old wildtype (WT), Fsp27−/−, leptin deficient (ob/ob) and leptin/Fsp27 double deficient (ob/ob/Fsp27−/−) mice (n = 3). (0.32 MB TIF) [file pone.0002890.s003.tif]

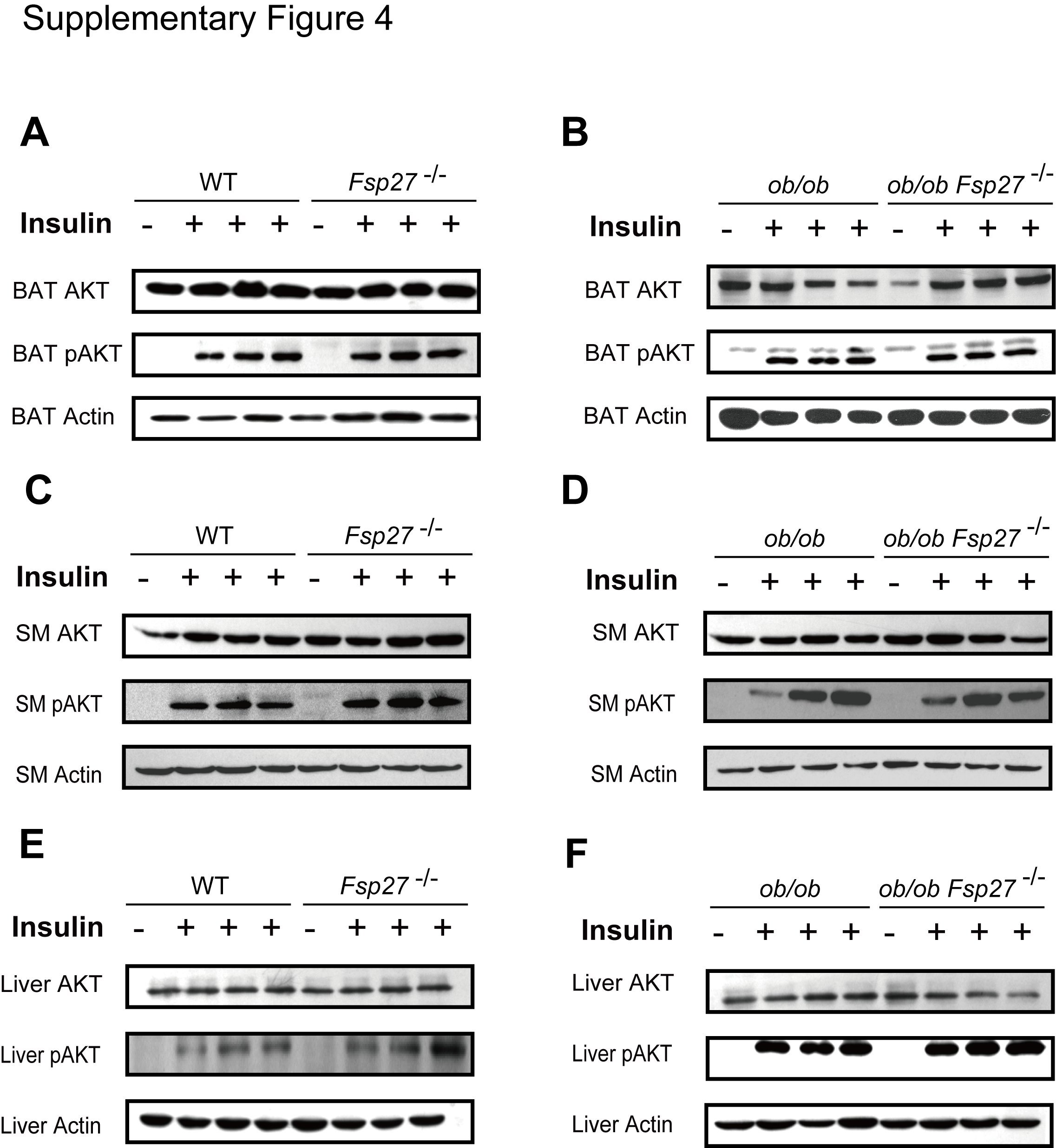

Supplement: Figure S4 — No difference of levels of AKT and phosphor-AKT in Fsp27−/−and ob/ob/Fsp27−/− mice. 3 months old mice that were fasted for 4 hours were intraperitoneally injected with 40mg/kg body weight of insulin. AKT proteins were immunoprecipitated with antibody against AKT and subsequently immunoblotted with antibodies again AKT or phosphor-AKT (pAKT). A, C& E. Western blot analysis for levels of total AKT and pAKT stimulated with and without insulin in BAT, skeleton muscle (SM) and liver of wild type (WT) and Fsp27 mutant (Fsp27−/−) mice. B, D& F. Western blot analysis for levels of total AKT and insulin stimulated pAKT in BAT, skeletal muscle (SM) and liver of ob/ob and ob/ob/Fsp27−/− mice. Actin was used as the loading control. (1.80 MB TIF) [file pone.0002890.s004.tif]

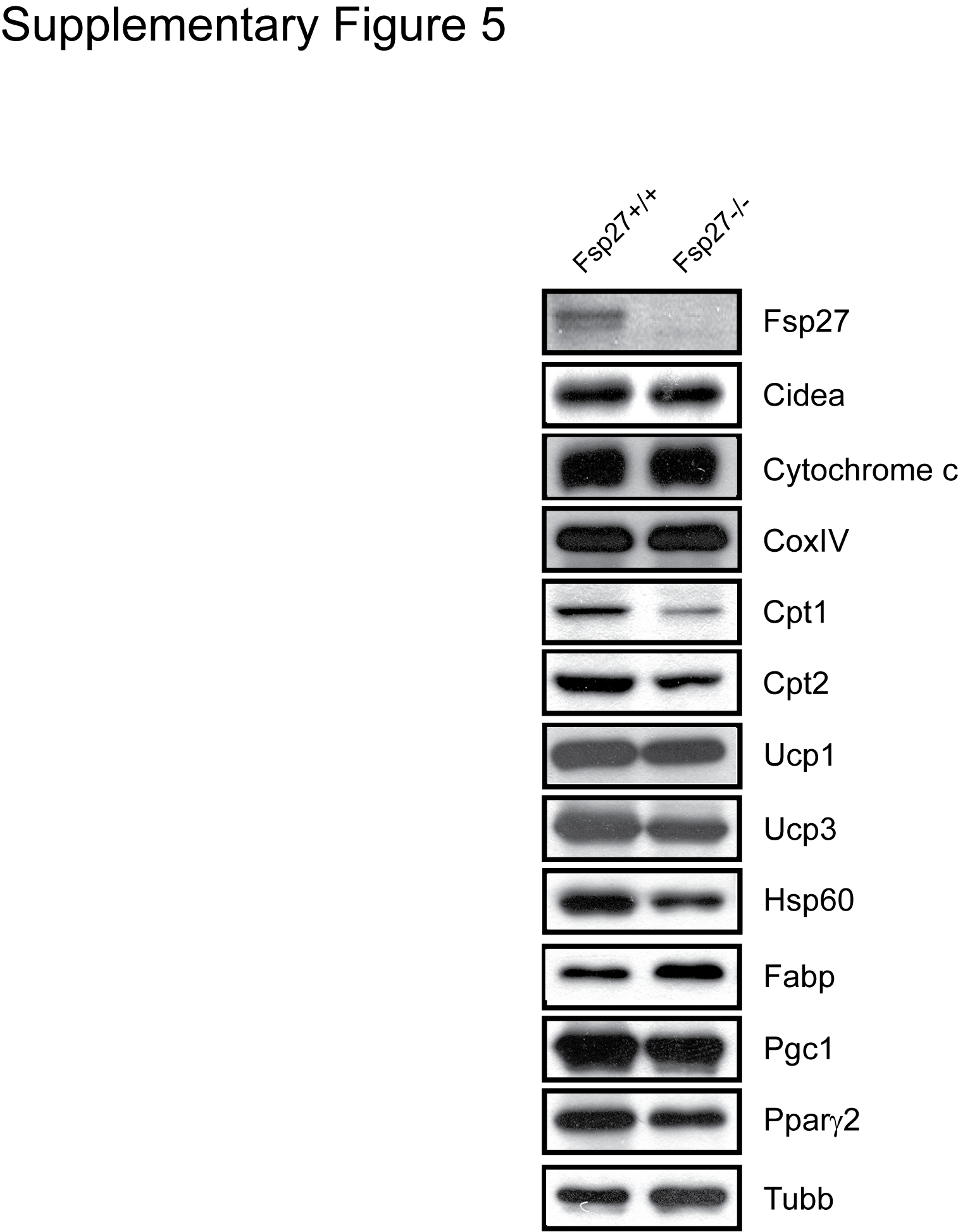

Supplement: Figure S5 — Western Blot analysis of total BAT tissue lysate from wildtype (Fsp27+/+) or Fsp27 null (Fsp27−/−) mice. β-tubulin was used as the loading control. Each panel is a representative of 4 individual experiments. Tubb represents tubulin. (1.03 MB TIF) [file pone.0002890.s005.tif]

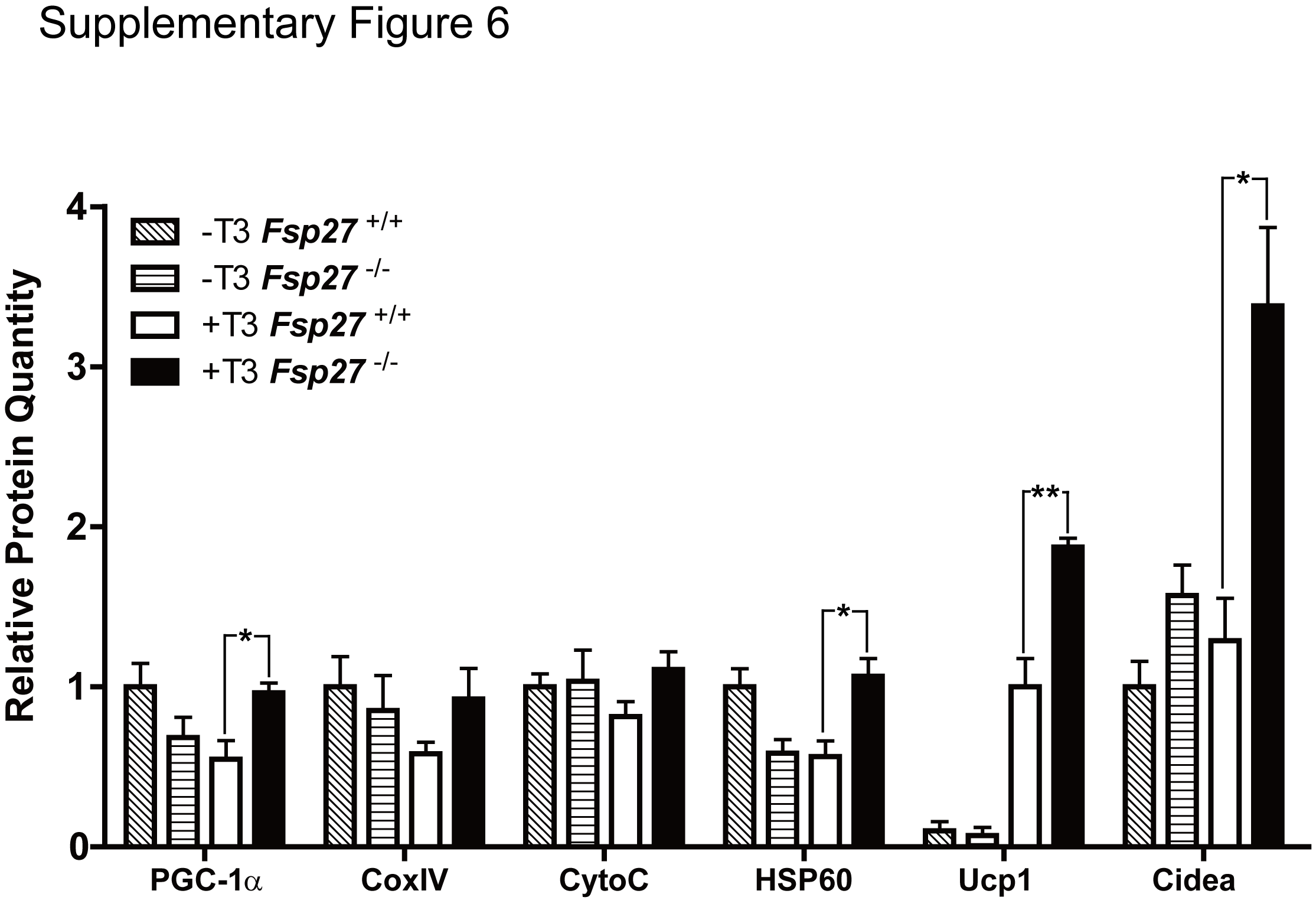

Supplement: Figure S6 — Densitometric reading of relative protein level in western blot analysis performed for the indicated protein of wildtype (Fsp27+/+) or Fsp27 null (Fsp27−/−) mice in Day 8 post-differentiated MEF cells with (+T3) or without (−T3) triiodothyronine (n = 3). (0.44 MB TIF) [file pone.0002890.s006.tif]
